# Yielding behaviour of active particles in bulk and in confinement

**DOI:** 10.1038/s41567-025-02843-7

**Published:** 2025-03-31

**Authors:** Yagyik Goswami, G. V. Shivashankar, Srikanth Sastry

**Affiliations:** 1 Theoretical Sciences Unit and School of Advanced Materials, Jawaharlal Nehru Centre for Advanced Scientific Research, Bengaluru, India; 2 Laboratory of Multiscale Bioimaging, Paul Scherrer Institute, Villigen, Switzerland; 3 Department of Health Sciences and Technology, ETH Zürich, Zürich, Switzerland

**Keywords:** Statistical physics, Biological physics

## Abstract

Collective behaviour in dense assemblies of self-propelled active particles occurs in a wide range of biological phenomena, including the dynamical transitions of cellular and subcellular biological assemblies such as the cytoskeleton and the cell nucleus. Here, motivated by observations of mechanically induced changes in the dynamics of such systems and the apparent role of confinement geometry, we show that the fluidization transition broadly resembles yielding in amorphous solids, which is consistent with recent suggestions. More specifically, however, we find that a detailed analogy holds with the yielding transition under cyclic shear deformation, for large but finite persistence times. The fluidization transition is accompanied by driving-induced annealing, strong dependence on the initial state of the system, a divergence of timescales to reach steady states and a discontinuous onset of diffusive motion. We also observe a striking dependence of transition on persistence times and on the nature of confinement. Collectively, our results have implications for biological assemblies in confined geometries, including epigenetic cell-state transitions.

## Abstract (web summary)

Assemblies of active particles display a range of dynamical phenomena. Simulations now show that the transition of an assembly of active particles from a jammed to a fluidized state is similar to the process of mechanical yielding seen in amorphous solids.

## Main

Active matter, composed of interacting self-propelled particles or entities, has been investigated intensely in recent years^1,2^, and displays novel aspects of dynamics and organization not realized in conventional condensed matter. Although avenues for synthesizing active materials have been well explored, many obvious examples of active matter arise in biological systems across length scales, from bird flocks and animal herds^3^, bacterial suspensions^4^ and tissues^5–7^ to the cytoskeleton^8^, which have been investigated both theoretically and computationally^9^ to comprehend the experimental findings. In several contexts, the systems of interest may be characterized as dense, disordered assemblies, displaying features characteristic of glassy systems or phenomenology associated with packings that undergo jamming.

In particular, novel behaviour arising in models of active glasses^5–7,10–21^ have been explored, seeking similarities with and departures from glass and jamming phenomenology in the absence of active forces or self-propulsion. In addition to investigations of the nature of relaxation dynamics, and approach to structurally arrested states with a decrease in thermal fluctuations and active forces and/or increase in density^5–7,13^, some recent works^14–18,21^ have addressed the converse problem of the loss of rigidity or fluidization, with an increase in the magnitude of active forces. Such a fluidization transition has an appealing analogy with the phenomenon of yielding in amorphous solids, on an increase in external driving through the application of, for example, shear stress or deformation, as discussed in detail elsewhere^14^. The active forces on individual particles, in the simple case where their orientations remain fixed, may be viewed as a spatially randomized generalization of shear-induced forces or displacements that arise when a global stress or strain is applied. Such an analogy has indeed been fruitfully pursued for the case in which the orientations of the active forces are fixed.

However, if one considers such active glass models in specific experimental contexts, including biological contexts in which active forces are generated by energy consumption, one must consider a situation in which the orientation of active forces is not fixed but may evolve in time—in a manner characterized by a persistence time. For active glasses with finite persistence times, we find that the fluidization transition bears striking similarities to the yielding transition under cyclic shear deformation, where the shear deformation is applied cyclically (typically) around the zero-strain state. The response of amorphous solids to cyclic shear is characterized^22,23^ by (1) annealing effects at small deformations, (2) strong dependence on the initial annealing state of the amorphous solids, (3) a discontinuous transition from an absorbing state with zero diffusivity of particles to a diffusive state with finite diffusivities and (4) diverging times to reach steady states as the yielding transition is approached from either side. As shown here, active glasses with finite (but large) persistence times show a transition from arrested to fluidized states, displaying these features remarkably.

In addition to the persistence time, we also study the role of confinement on the transition to the fluidized state. The presence of a confining boundary has been found to induce non-trivial behaviour in active systems^24–26^. Such effects can be expected to play an important role in determining the properties of many biological assemblies that experience strong confinement conditions. A particular example of this kind is the organization of chromatin within the nucleus, where an interplay of active forces and the effect of confinement boundary are of potential interest to understand in the context of mechanically induced cell-state changes^27–29^. Although we do not attempt to model the specific details of such biological assemblies in this work, the results we obtain should be of relevance in several such contexts.

Instead, we aim to study an idealized system, using which we aim to comprehend the role of perturbing factors that may be biologically relevant. We, thus, consider the arrested-to-fluidized (or yielding) transition in a dense assembly of particles at very low temperatures, subjected to active forces that have a finite persistence time. We investigate the yielding behaviour of local-energy-minimum configurations, referred to as glasses or amorphous solids, which are prepared with different degrees of annealing (Methods and Supplementary Fig. 1), followed by local energy minimization, so that their initial energies vary over a wide range. We simulate a two-dimensional (2D) 65:35 (Kob–Andersen) binary mixture of particles interacting with the Lennard–Jones potential, whose dynamics is described by the Langevin equation, with forces from interparticle interactions, and active forces whose orientation changes diffusively with a specified persistence time. Further details of the model and simulation methods are given in the Methods.

We first investigate the yielding behaviour as a function of the strength of activity and persistence time, and demonstrate that the yielding behaviour indeed strikingly resembles the yielding transition under cyclic shear. Since we also expect the effects of confinement to be important, we consider different confinement geometries, where the interacting particles experience short-range attractive interactions and strong repulsion at smaller distances to the confining boundary.

## Results

We first consider a system of *N* = 1,000 particles in two dimensions with a number density of *ρ* = 1.2, a small reduced temperature of *T* = 10^−3^ and periodic boundary conditions. Each of these cases is simulated using Langevin dynamics, with forces arising from interparticle interactions and an active force, with a time step of d*t* = 0.01*τ*, over a range of values of the active force magnitudes *f*, which reorients on a timescale given by the persistence time *τ*_p_ (Methods). We, thus, present our results for a fixed value of *τ*_p_ as a function of *f*, considering amorphous solids with a range of initial energies as described earlier (Supplementary Fig. 1). In all cases, the systems evolve to reach steady states, and we report the properties of the system in the steady state (unless otherwise stated).

### Yielding transition

**Fig. 1:**
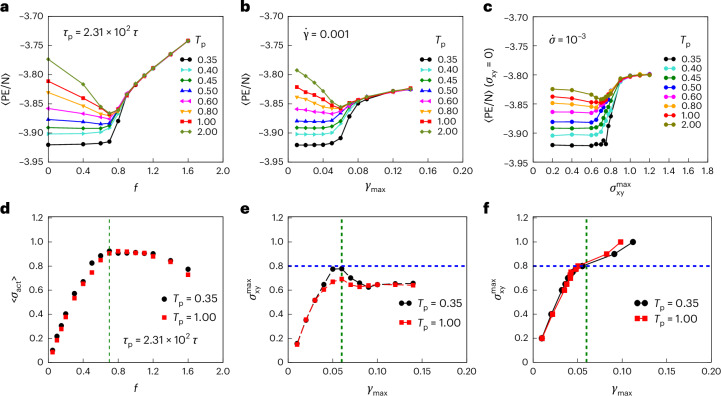
Yielding diagram under active driving. **a**, Amorphous solids subject to active forces display annealing to lower energies for high-energy initial configurations for small active forces, with the degree of annealing diminished or absent for lower-energy initial states. The average steady-state energies, 〈PE/N〉, are shown. A common, ergodic, fluidized state is observed above a critical value of active forces. These observations are analogous to those observed for yielding under cyclic shear deformation. **b**,**c**, Steady-state energies for energy at zero strain in strain-controlled cyclic shear (**b**) and at zero applied shear stress in stress-controlled cyclic shear simulations (**c**). **d**, Yielding transition is accompanied by a saturation of the active stress, computed as explained in the Methods. **e**, Measured shear stress ${\sigma }_{xy}^{{\rm{max}}}$ at strain amplitude *γ*_max_ for strain-controlled cyclic shear. The vertical dashed green line is an estimate of the yield strain and the horizontal dashed blue line is an estimate of the yield stress. **f**, Applied maximum shear stress ${\sigma }_{xy}^{\max}$ versus the measured value ${\gamma }_{\max}=\gamma ({\sigma }_{xy}={\sigma }_{xy}^{\max})-\gamma ({\sigma }_{xy}=0)$ in stress-controlled cyclic shear simulations.

**Fig. 2:**
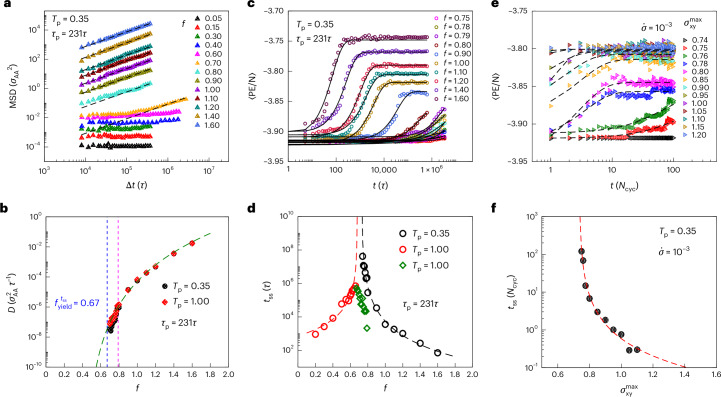
Diffusivity and time to reach the steady state. **a**, MSD for different magnitudes of the active force showing a transition from an absorbing (non-diffusive) to a diffusive state for the well-annealed case. The black dashed lines are linear best fits to the data. **b**, Change in diffusivity with the active force magnitude, for both poorly annealed and well-annealed samples, shown for values of the active force for which the MSD shows a diffusive regime within the minimum expected waiting time for particles to escape the cage of their local neighbourhood (Supplementary Information). The diffusivity data for *f* > 0.8 are fit to a power law (dashed magenta vertical line indicates the lowest *f* for which the diffusivity data are included in the fitting procedure), with data for *f* < 0.8 in reasonable agreement with the extrapolation from the fit. The dashed blue vertical line marks the value of *f* at which *t*_ss_ goes through a maximum in **d** for the poorly annealed case. The extrapolation of the diffusivity data indicates a vanishing diffusivity at *f* = 0.43. **c**, Relaxation curves for energy versus active force, obtained by averaging over eight independent trajectories in logarithmically spaced time intervals. **d**, Relaxation times *t*_ss_ exhibiting divergence at the yielding transition. The dashed black and red lines are the best fits to the data. **e**, Relaxation curves for potential energy versus number of cycles for stress-controlled cyclic shear simulations, obtained by averaging over 16 independent trajectories in logarithmically spaced time intervals. **f**, Relaxation times, measured from the fluidization time for well-annealed samples, exhibiting divergence at the yielding transition (the dashed red line is the best fit to the data) with *σ*_yield_ = 0.735 and exponent *β* = 1.87 (Supplementary Information provides further discussion on exponents).

In cyclic shear simulations, the key characteristics of the yielding transition that have been highlighted in previous studies are as follows. (1) Annealing effects: it is found that for high initial energies (or poorly annealed amorphous solids), the final (non-diffusive or absorbing) steady states exhibit progressively larger degrees of mechanically induced annealing (a decrease in the internal energy) with increasing amplitude of strain (the control parameter, like the strength of active force *f* in the present case) till the yielding or fluidization transition. By contrast, well-annealed (low-initial-energy) amorphous solids exhibit little annealing before—and strongly discontinuous change at—the yield point. (2) Dependence of transition on the degree of initial annealing: the transition is substantially more or less sharp depending on the degree of initial annealing. (3) Discontinuous change: there is a discontinuous onset of diffusive behaviour and a drop in stress across the yielding transition. (4) Divergence of timescales: the time to reach the steady states diverge at the transition, measured by the time taken for a fluidized steady state to be achieved for large-amplitude strain beyond the yield point, or a localized or absorbing state to be reached, below the yield point. We, thus, consider the corresponding behaviour of the steady-state energies and stresses (Fig. 1) and diffusion and timescales (Fig. 2) for the active glasses we now investigate. Figure 1a shows, as a function of the active force strength *f*, the final (steady-state) energies reached, for a number of initial energies, for *τ*_p_ = 2.31 × 10^2^*τ*. The resulting curves bear striking similarities with the cyclic shear yielding diagram^23^; here, as a function of the strain amplitude, (1) poorly annealed (high-energy) glasses show progressive mechanical annealing before yielding and (2) well-annealed glasses show strong, discontinuous yielding. In Supplementary Fig. 2, we show the corresponding steady-state energies at two other, higher, values of *τ*_p_.

The stress can be computed from the first derivative of energy with respect to strain, and the corresponding procedures for computing the active stress (for example, refs. ^16,18^) require identifying a strain step Δ*γ* associated with displacements in the presence of active forces. Following ref. ^18^, we first consider that the alignment of particle velocities with the direction of active force produces a net strain rate (equation (5) and Supplementary Information), which can be used to obtain the strain step Δ*γ* over a time interval (Methods). We analyse the parametric dependence of Δ*E* on Δ*γ* to compute the active stress. The resultant average stress 〈*σ*_act_〉, as defined in equation (6), is found to increase linearly with *f* until the yield point at which it attains the maximum value (Fig. 1d). The net velocity alignment, from which we determine an effective strain rate, remains negligible in magnitude below the yield point, with a subsequent increase for active forces (or applied shear stresses in stress-controlled cyclic shear simulations) larger than the yield value (Supplementary Figs. 3–5), as also noted in similar contexts^18,30,31^.

For comparison, we show the steady-state energies for cyclic shear, where the control parameter is either the strain amplitude (Fig. 1b) or the amplitude of applied shear stress (Fig. 1c). We note that the increase in energies in the post-yield regime is more pronounced for the active case, compared with that in the applied strain/stress case. The reason is that in the case of applied strain/stress, we consider energies, stroboscopically, that is, at strain *γ*_*xy*_ = 0 or stress *σ*_*xy*_ = 0. The corresponding energies are lower compared with an average over the cycle^22^, and more so for larger amplitudes of strain/stress. In the case of active dynamics that we implement, we have no analogue of stroboscopic configurations; therefore, we average the energies over time, leading to the observed differences. The non-monotonic dependence of energies with active driving, and the analogy to cyclic shear was previously addressed^14^, but with explicit ‘back-and-forth’ cyclic self-propulsion. Similar results have also been reported recently for active ‘run-and-tumble’ driving^32^. In Fig. 1e, we show the measured shear stress at the maximum strain. The measured shear stress (Fig. 1e) is computed from the virial expression and is compared with that obtained from the procedure outlined above for active stress in Fig. 1d (Supplementary Fig. 6). The close agreement validates the latter procedure, although improvements to the procedure merit further investigation. In Fig. 1f, we show the maximum applied shear stress versus the measured strain amplitude in cyclically sheared systems in which the stress is controlled. Comparing the results in Fig. 1d with those in Fig. 1e,f, we note that although the computed stresses clearly mark the fluidization transition for the active case, the stress overshoot, the presence of a constant flow stress and the difference between the peak stresses of the well-annealed samples and the poorly annealed samples is less pronounced for active driving as well as its direct analogue in cyclic shear—the stress-controlled case. Moreover, in the case of both active driving and stress-controlled cyclic shear, the difference in the elastic branch between the poorly annealed and well-annealed sample appears to be minimal.

### Onset of diffusive dynamics and relaxation to the steady state

We next consider the mean squared displacements (MSDs) of particles as a function of elapsed time, in the steady state, for different values of *f* (Fig. 2). It can be seen that the MSD curves exhibit a saturation below a critical value of *f* (which can be deduced from the data shown in Fig. 1) and a linear dependence on time for higher *f* values. The corresponding diffusion coefficients shown in Fig. 2b jump discontinuously at the yield point, analogously with cyclic shear yielding, indicative of an absorbing-to-diffusive transition (point (3) discussed in the ‘Yielding transition’ section). The diffusivity data in Fig. 2b are fit to a power law for *f* > 0.8 (magenta vertical line), to identify a value of *f* at which the diffusivity is extrapolated to vanish. Diffusivities for *f* in the window 0.7 ≤ *f* ≤ 0.8 adhere to the extrapolated fit, though they are not used to obtain the fit itself. The MSDs for *f* < 0.7 (*T*_p_ = 0.35) and *f* < 0.65 (*T*_p_ = 1.00) clearly do not follow a *t*^1^ scaling and show strong indications of structural arrest (Supplementary Fig. 7; further analysis is provided in Supplementary Figs. 8 and 9). Thus, diffusivities cease to be meaningfully defined at *f* values close to the extrapolated divergence of *t*_ss_ (Fig. 2b, dashed blue vertical line), whereas the extrapolation of diffusivities post-yield to smaller *f* suggests a vanishing diffusivity at much smaller values of *f* = 0.43.

We next consider the behaviour of timescales around the yield point (point (4) discussed in the ‘Yielding transition’ section), which shows a divergence for cyclic shear yielding in which the strain amplitude is the control parameter. To obtain the corresponding data for active driving, we consider the relaxation of energy (shown in Fig. 2c for *T*_p_ = 0.35) for different values of *f*. A stretched exponential fit to the average energy over time yields a relaxation timescale 〈*t*_ss_〉 (equivalently, a fluidization timescale for the well-annealed case; Supplementary Information), which is shown in Fig. 2d, indicating that the time to approach the steady state becomes progressively longer as the yielding transition is approached from either side, with an apparent divergence at the transition. For the poorly annealed samples (*T*_p_ = 1.00), data for force magnitudes both smaller and larger than the critical force are shown, with a peak strongly evident in the vicinity of the yield point. For the case of *T*_p_ = 0.35, only the larger forces are shown. The estimated times to steady state obtained from the procedure described above compare well with the corresponding estimates from the average first passage time to reach the steady-state energy (Supplementary Fig. 10). Here *t*_ss_ is fit to a form *B*/(*f* – *f*_yield_)^*β*^, with a value of *β* = 3.984 and *f*_yield_ = 0.699 obtained from the fits for the well-annealed case (Fig. 2d). For the poorly annealed case (*T*_p_ = 1.00), data for force magnitudes lower than that at which *t*_ss_ goes through a maximum are shown in red, whereas data for larger forces are shown in green. For *f* less than the estimated critical force, *β* = 2.607 and *f*_yield_ is bounded from above, while fitting, at the lowest *f* value at which the non-monotonicity in *t*_ss_ appears.

We similarly analyse the relaxation of the average energy over time for well-annealed samples, prepared at *T*_p_ = 0.35, when subjected to cyclic shear with the applied shear stress and show these in Fig. 2e. Stretched exponential fits yield the relaxation timescale 〈*t*_ss_〉, which similarly diverge as the yielding transition approaches (Fig. 2f shows the well-annealed case). For stress-controlled cyclic shear, *β* is estimated at 1.87, substantially different from the value for active driving, but similar to that reported for strain-controlled cyclic shear^33^. *t*_ss_, $\dot{\gamma }$ and the control parameters (*f* or ${\sigma }_{xy}^{max}$) have been shown in the literature to be closely related, with the well-known Herschel–Bulkley relation governing the relationship between $\dot{\gamma }$ and the control parameter. Such a relationship with the strength of active driving as the control parameter was investigated in ref. ^14^ and subsequently in other works^18^. We study the relevant exponents for the case of active driving and strain- and stress-controlled shear in greater detail in the Supplementary Information (Supplementary Figs. 3–5 show the fits) and find that the Herschel–Bulkley exponents for both modes of driving have similar values, which are also in the range of those reported for 2D systems in the literature.

The above results clearly demonstrate that yielding in a system with active forces with a finite persistence time has the characteristics of yielding under cyclic shear. Intuitively, a way to understand this is to note that in addition to having no macroscopically well-defined direction of driving as in, say, simple shear deformation, with finite persistence time, the direction of driving also changes over time, thereby bearing a broad similarity to the back-and-forth driving under cyclic shear.

In the thermal limit of low persistence times *τ*_p_, the active driving we consider is equivalent to a higher effective temperature, with the contribution from active driving increasing quadratically with *f* (Supplementary Figs. 11 and 12 show a comparison with kinetic energies in the low persistence limit and passive dynamics). Extensions of such a relationship to finite and large persistence times have also been discussed^13,20,34^. We also perform passive thermal dynamics in the constant-temperature and constant-volume ensemble for temperatures at which the samples undergo fluidization. Interestingly, we find (Supplementary Fig. 13) that such ‘heating’, although showing a change from absorbing to fluidized states across a melting temperature *T*_melt_, neither exhibit a diverging timescale to the steady state nor a clear jump in the diffusivity across *T*_melt_. We next focus on the dependence of this yielding transition on parameters that may be of relevance not only in the biological context of our interest but more generically. We, thus, consider the effect of the change in persistence times, and the nature of confinement, on the yielding transition.

### Role of persistence time in the yield point

**Fig. 3:**
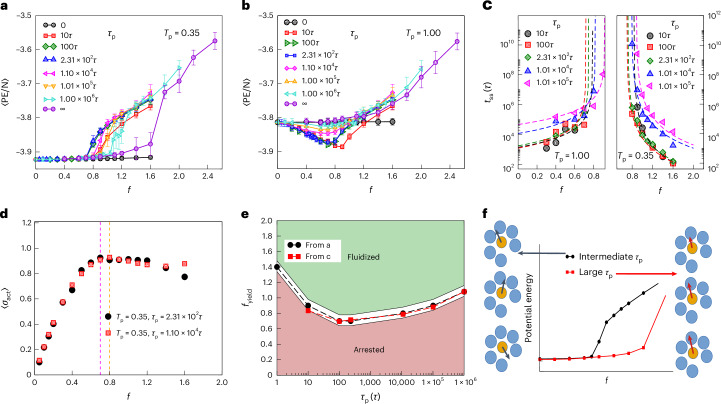
Dependence of yielding behaviour on persistence time. **a**,**b**, The yielding transition shifts to smaller force magnitudes *f*, as the persistence time increases from *τ*_p_ = *τ* to 100*τ*–200*τ* and thereafter to larger *f* as the persistence time increases further, for the well-annealed (**a**) and poorly annealed (**b**) initial configurations. The mechanical annealing in the latter case diminishes with an increase in the persistence time. Data are obtained by averaging over eight independent trajectories. The error bars in **a** and **b** denote the standard deviations at the respective *f*. **c**, Divergence of the time to reach the steady state at different persistence times. The dashed lines are best fits with the exponent fixed based on the data for *τ*_p_ = 2.31 × 10^2^*τ*. **d**, Stress as a function of active force, with a shift in the value of force (*f*) at which deviation from linearity first occurs. The value of *f* corresponding to the peak stress is indicated by a vertical magenta line for *τ*_p_ = 2.31 × 10^2^*τ* and a vertical orange line for *τ*_p_ = 1.01 × 10^4^*τ*. **e**, Yielding transition force, measured from the departure of the potential energy from the initial preparation value in **a** and from the extrapolated divergence of the timescale to the steady state in **c**, shown as a function of *τ*_p_. **f**, Schematic of the role of persistence time in the intermediate-to-high-persistence-time regime. Intermediate persistence times facilitate the rapid exploration of routes to escape the cages constituted by their nearest neighbours, leading to yielding at small active driving magnitudes. As the persistence time increases, this capacity for exploration decreases and particles instead ‘break through’ their cages, which requires large-magnitude active forces.

A key control factor of relevance in various biological contexts is the persistence time, which determines the timescale over which the active force reorients. Changes to the persistence time have strong implications for the efficacy of the exploration of phase space, producing different dynamical regimes^13,19,20,31,35,36^, motivating us to study changes in yielding behaviour arising from changes in the persistence time. We study the effect of changing the persistence time on mechanical annealing below and on the location of the yield point for both well-annealed (Fig. 3a) and poorly annealed (Fig. 3b) samples. Interestingly, we observe that the yielding transition point *f*_yield_ exhibits non-monotonic behaviour as the persistence time increases, decreasing with increasing persistence until *τ*_p_ ≈ 200*τ*, and increasing thereafter. For the poorly annealed case, the mechanical annealing effects consistently diminish as the persistence time increases. In both cases, the apparently continuous increase in energies in the yielded branch have to do with the difficulty in reaching the steady state close to the transition, as explained further in the Supplementary Information (Supplementary Fig. 14). We also show the results for the limit of zero persistence time (*τ*_p_ = d*t*, as mentioned above), which reveal a negligible change in the range of *f* values shown (which correspond to very small increments in the bath temperature; Supplementary Figs. 11 and 12). In Fig. 3c, we show the change in *t*_ss_ as the persistence time increases, where *t*_ss_ values below the yield point are obtained for poorly annealed initial samples and above the yield point for the well-annealed samples, as before (Supplementary Figs. 14 and 15 show the fits to extract *t*_ss_). The points of divergence of *t*_ss_ shift with *τ*_p_ non-monotonically, consistent with the results shown in Fig. 3a,b. Figure 3d shows the active stress *σ*_act_, which increases up to the yield point (Fig. 1b), which are slightly different for the two cases shown. In Fig. 3e, we summarize the dependence of the yield point on *τ*_p_ from the data in Fig. 3a,c, highlighting the consistent non-monotonicity in the yield point. Such non-monotonicity with increasing persistence time has also been reported elsewhere in similar contexts^37^. We understand the trends in *f*_yield_ with *τ*_p_ in the context of the changing nature of dynamics with *τ*_p_, particularly with reference to the thermal limit at very small *τ*_p_. At small persistence times (*τ*_p_ < 100), increasing the persistence time shifts the nature of dynamics away from the thermal limit and leads to a decrease in the yield point, consistent with previous work^13^. At very high persistence times, the particles are more readily ‘jammed’ in kinetically arrested higher-energy states, reminiscent of the phenomenology explored in other work^13,19,20^, and consistent with the data and discussion in refs. ^13,20^ (Supplementary Fig. 16). The schematic shown in Fig. 3f illustrates how the efficiency of the exploration of phase space and, in turn, the activity-induced annealing and the yield point may be altered by the persistence time, in the regime of intermediate to high persistence times. In this regime, the role of high persistence times is analogous to shear rates (or, equivalently for cyclic shear, high frequencies), in that both large persistence time or shear rates/frequencies, in the respective cases, correspond to large Péclet numbers: ${\rm{Pe}}=\dot{\gamma }{\tau }_{\alpha }$ for shear^38^, where *τ*_*α*_ is the relaxation time, and ${\rm{Pe}}=\frac{f{\tau }_{{\rm{p}}}}{\zeta \sigma }$ for the active case we consider^13^, where *ζ* is the dissipative friction coefficient and *σ* is the particle size; for cyclic shear, the shear rate computed at zero strain is $\dot{\gamma }={\gamma }_{max}\omega$, where *ω* is the frequency and *γ*_max_ is the amplitude. Such a picture is not expected to hold for small *τ*_p_ values, where the system begins to resemble a thermal system as the *τ*_p_→0 limit is approached. Our results, thus, suggest the observed non-monotonicity of *f*_yield_ to be a consequence of transitioning from the low-persistence-time regime in which active forces contribute to an increased effective temperature to the large-persistence-time regime in which active driving leads to yielding, analogous to driving by external stresses.

### Simulations in confinement

We next consider the influence of confinement geometry on the yielding behaviour, a factor that may be relevant in several, including biological, contexts. For example, experiments studying the dynamics and spatial organization of chromatin within the nucleus have pointed to the important role of confinement geometry in cells subjected to mechanical conditions in which the nuclear membrane exhibits large-scale fluctuations, which are persistent on timescales an order of magnitude or more larger than the timescales of the diffusive motion of fluorescently labelled regions of chromatin^28,39^. Such driving-induced nuclear deformations have been implicated in cell-state transitions^40,41^, pointing to the role of spontaneous driving, far from the aforementioned low-persistence-time thermal limit, in biologically relevant assemblies.

Active particles in confinement have previously been noted to display rich behaviour such as wall aggregation^24,25^ and the formation of cavities that are otherwise also formed in bulk active systems undergoing motility-induced phase separation. Of particular interest are the curvature-induced changes in the density of accumulation^25,42^, and the generation of large-scale rotational flows^43^, strongly dependent on the curvature at the boundaries^44^. We consider here a case of high density and investigate the yielding behaviour as the strength of active driving increases. We compare the yielding behaviour for two confinement geometries: (1) a nearly circular confinement with aspect ratio (ratio of major axis to minor axis) *e* = 1.2 and (2) a strongly elliptical confinement with *e* = 1.8. Particles experience attractive interactions of the Lennard–Jones form with the wall, with the parameters as given in the Methods. Initial configurations with different annealings are prepared by a procedure analogous to the bulk case (Methods and Supplementary Fig. 18). These configurations are then subjected to active forces, at a persistence time of *τ*_p_ = 200*τ* and the potential energy is tracked to identify a transition from an absorbing to a diffusive state (Supplementary Fig. 19).

**Fig. 4:**
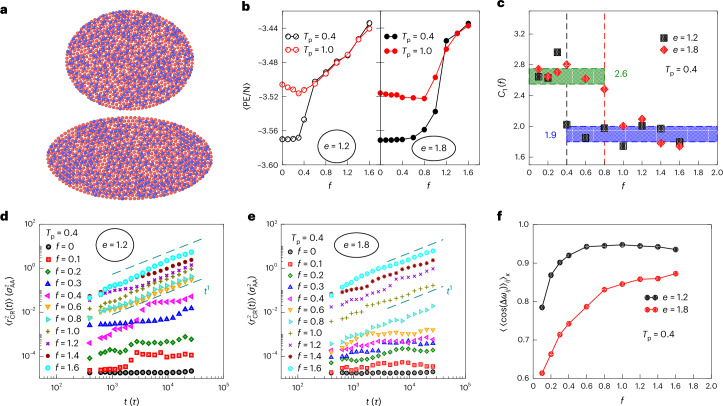
Dependence of yielding behaviour on confinement. **a**, Simulations are performed in the two confinement geometries, with eccentricities of *e* = 1.2 (more isotropic) and *e* = 1.8 (more anisotropic). Red (blue) discs represent A-type (B-type) particles. **b**, Yielding diagram with the steady-state potential energy per particle at different values of the active force in the two geometries, comparing the behaviour for a well-annealed case (*T*_p_ = 0.4) with that of a poorly annealed case (*T*_p_ = 1.0). **c**, Static correlation length scale *C*_l_ identified from the decay of the wall-particle density correlation function (Supplementary Information) for the two geometries simulated from well-annealed initial samples. The green-shaded (blue-shaded) region shows the typical values for *C*_l_ in the pre-yield (post-yield) state for either geometry. The vertical dashed black line marks the estimated critical *f* value for *e* = 1.2 and the vertical dashed red line shows the critical *f* value for *e* = 1.8. **d**,**e**, ${r}_{CR}^{2}(t)$ values are shown for *e* = 1.2 (**d**) and *e* = 1.8 (**e**) for *T*_p_ = 0.4—the well-annealed case. Note that in the absorbing regime of low force, ${r}_{CR}^{2}(t)$ remains below 0.01, indicating an average displacement of ~10% of *σ*_AA_ for each particle with respect to its caging neighbours. The dashed teal lines in **d** and **e** indicate a linear dependence characteristic of the diffusive regime. **f**, Alignment of the motion of particles near the boundary with that of their nearest neighbours, summed over the range of curvature values, *κ* (equation (10)), in the two geometries. The alignment saturates to high values on yielding and is low in the pre-yielded state, for either geometry.

The geometries considered and the resulting yielding behaviour is shown in Fig. 4a. The results indicate that in going from a more circular to a more elliptical geometry, the yielding point is pushed to larger values of the active force. In both geometries, the post-yield states are ergodic, as observed from the dependence of the potential energy per particle on *f* (Fig. 4b). Further, we analyse the correlation of particle density as a function of distance from the confinement boundary along the normal to the boundary (Supplementary Information; Supplementary Fig. 20 shows the representative plots of *g*_w_(*r*)). We also identify a length scale of correlation *C*_l_ (Fig. 4c), showing a higher correlation length scale of approximately 2.6*σ*_AA_ in the pre-yield state for both geometries and a sharp decrease to a value of approximately 1.9*σ*_AA_ for both geometries, across the respective estimated yield points of *f* ≈ 0.3 for *e* = 1.2 and *f* ≈ 0.8 for *e* = 1.8. Although *C*_l_ is not negligible compared with the confinement dimensions, one observes largely similar values for either geometry in both pre-yield and post-yield states, with a sharp change across the respective yield points. It has been observed that confined active systems exhibit global rotations^43^, which should be subtracted in computing diffusive displacements of particles. This is accomplished, following ref. ^45^, by computing the cage-relative MSDs $\langle {r}_{{\rm{CR}}}^{2}(t)\rangle$ (Methods; Fig. 4d shows the more circular case and Fig. 4e shows the more elliptical case) starting from well-annealed samples. The superposition of global rotations and local rearrangements results in a less reliable measure of diffusive motion despite the correction used. Nevertheless, one observes that the system remains in a non-diffusive absorbing state in the pre-yield regime, whereas diffusive motion sets in beyond the yielding *f* value in each of the two geometries, *f*_yield_ ≈ 0.3 for *e* = 1.2 and *f*_yield_ ≈ 0.8 for *e* = 1.8 (Supplementary Video 1 shows the large-scale rearrangements for *e* = 1.2 at *f* = 0.6 over a time window and Supplementary Video 2 shows the arrest for *e* = 1.8 at the same value of *f* = 0.6; Supplementary Information provides the captions for Supplementary Videos 1 and 2). We further observe a distinct change from a strongly patterned time- and run-averaged 2D density histogram, indicative of arrested states, to a more homogeneous 2D density histogram, indicative of flowing, ergodic states across the respective transitions (Supplementary Fig. 21). Such a large change in the yield value of the active force is not readily explained by the differences in perimeter length, of about 6%, differences in the typical static correlation length scales *C*_l_, or to differences in the dimensions (minor- and major-axis lengths of 14.87*σ*_AA_ and 17.84*σ*_AA_ for *e* = 1.2 and *e* = 12.13 and 21.834*σ*_AA_ for *e* = 1.8) and point to a sensitive dependence of the fluidization transition on the confinement geometry. In Fig. 4f, we show the dependence of the alignment of particle motion with that of the nearest neighbours, over a finite time window, near the confinement boundary (Methods). Regions of high curvature in confined active systems have been shown to result in accumulation for non-interacting particles, as well as in substantial changes to the degree of alignment of particle motion^25^ (Supplementary Fig. 22 shows the curvature dependence of motion alignment). In a similar vein, though differing in detail, we find that particle motion near the boundary is aligned to a much higher degree for the less elliptical geometry, and that alignment decreases substantially below the critical *f*_yield_ value in both geometries. Such dependence, including the role of wall interactions, needs to be explored in greater detail.

These results show that at a given magnitude of active forces, a change to a more symmetric geometry can induce yielding and fluidization. They highlight the role of confinement geometry in state transitions in assemblies subject to active forces, including the biological assemblies mentioned above, as discussed briefly below.

## Discussion

Our results demonstrate the existence of a strong analogy between cyclically sheared amorphous assemblies and actively driven assemblies. Both modes of drive result in a yielding transition from an absorbing state to a fluidized state beyond a threshold. We find a strong correspondence in the yielding phase diagram, with those produced for stress-controlled and strain-controlled cyclic shear, having regimes of annealing and yielding to an ‘ergodic’ state (point (1) discussed in the ‘Yielding transition’ section), (2) a similar dependence on the initial state of the amorphous solids, (3) the onset of a diffusive state for large perturbations and (4) the divergence in the time to reach the steady state.

Active driving with a finite persistence time, and in confinement, as implemented in this work, is motivated by our interest in biological systems that display a transition from rigid to flowing states when subjected to analogous mechanical conditions. Our results demonstrate that *τ*_p_ is indeed a key parameter, with a non-monotonic dependence of the critical yield force *f*_yield_ on the persistence time. We also find that changing the confinement geometry leads to a change in the yielding behaviour, with the transition to fluidized states occurring at much lower values of the active force for the more isotropic confinement geometry. That confinement geometry alone affects the yielding behaviour in such a manner is, in our view, an interesting effect with numerous possible implications in various contexts. Although the model system we investigate does not serve as a model for specific biological assemblies, we believe that the results are of interest to contexts such as the organization and dynamics of chromatin in the nucleus. In such contexts, the interplay and complex causal relationships of the nature of active driving, confinement and fluidization state are important to elucidate for developing faithful theoretical models. The analysis presented here, we believe, forms an important first step in that direction.

## Methods

### Model—2D binary mixture

We simulate a bidisperse Lennard–Jones mixture at constant *N* and *V* with periodic boundary conditions (wraparound), at high density *ρ* = 1.2 in two dimensions. The interaction is truncated at a cut-off of ${r}_{{\rm{c}}}^{\alpha \beta }={\sigma }_{\alpha \beta }$, where *α* and *β* denote the type of interacting particles *i* and *j*, either A or B, and is given by

$${\phi }_{{\rm{LJ}}}({r}_{ij})=\left\{\begin{array}{l}4{\epsilon }_{\alpha \beta }\left[{\left(\frac{{\sigma }_{\alpha \beta }}{{r}_{ij}}\right)}^{12}-{\left(\frac{{\sigma }_{\alpha \beta }}{{r}_{ij}}\right)}^{6}\right]\quad \,\text{if}\,\quad {r}_{ij}\le {r}_{{\rm{c}}}^{\alpha \beta }\quad \\ =0\quad \,\text{otherwise}\,\quad \end{array}\right.$$

We choose *N* = 1,000 particles in a 65:35 mixture with *N*_A_ = 650. Reduced units are used throughout, in terms of *σ*_AA_, *ϵ*_AA_ and the particle mass *M*, with the unit of time $\tau =\sqrt{M{\sigma }_{{\rm{AA}}}^{2}/{\epsilon }_{{\rm{AA}}}}$, *σ*_AB_/*σ*_AA_ = 0.8, *σ*_BB_/*σ*_AA_ = 0.88, *ϵ*_AB_/*ϵ*_AA_ = 1.5 and *ϵ*_BB_/*ϵ*_AA_ = 0.5.

### Initial sample preparation

We perform constant-temperature (*NVT*) molecular dynamics simulations using the Nosé–Hoover thermostat with a time step of d*t* = 0.01 in the large-scale atomic/molecular massively parallel simulator (LAMMPS) software suite^46^ at different preparation temperatures *T*_p_ to generate equilibrated liquid configurations. Energy-minimum configurations (inherent structures) are produced by performing conjugate gradient minimization using LAMMPS. The inherent structure energies show a *T*_p_ dependence (Supplementary Information).

### Shear simulations

Cyclic shear simulations are performed at a finite shear rate and temperature, propagating the SLLOD equations of motion using the LAMMPS software suite^46^. Simulations are performed with a time step of d*t* = 0.01, with the strain being varied sinusoidally, for a 100 cycles for each strain amplitude *γ*_max_. The strain rate, computed at zero strain, is $\dot{\gamma }=0.001$.

### Equations of motion for active dynamics

Active dynamics are performed starting from the energy-minimized configurations by integrating the following stochastic equations of motion.

1 $$\begin{array}{rcl}{\dot{{\bf{p}}}}_{i}&=&-\zeta {{\bf{p}}}_{i}+\mathop{\sum }\limits_{j\ne i=1}^{N}{{\bf{f}}}_{{\bf{ij}}}+f\,{\hat{{\bf{n}}}}_{i}+{{{\mathbf{\xi }}}_{{\bf{t}}}}^{i}\\ {\dot{{\bf{x}}}}_{i}&=&{{\bf{v}}}_{i}\equiv {M}^{-1}{{\bf{p}}}_{i}\\ {\dot{{\mathbf{\theta }}}}_{i}&=&{{{\mathbf{\xi }}}_{{\bf{r}}}}^{i}\end{array}$$

The translational noise *ξ*_t_ has zero mean and variance given by $< \,{\xi }_{{\rm{t}}}^{\,i\alpha }(t)\,{\xi }_{{\rm{t}}}^{\,j\beta }({t}^{{\prime} })\, > =2{k}_{{\rm{B}}}TM\zeta {\delta }_{ij}{\delta }_{\alpha \beta }\delta (t-{t}^{{\prime} })$. The rotational diffusion of the direction of active forcing similarly has zero mean and variance given by $< {\xi }_{{\rm{r}}}^{\,i}(t)\,{\xi }_{{\rm{r}}}^{\,j}({t}^{{\prime} }) > =2{\tau }_{{\rm{p}}}^{-1}{\delta }_{ij}\delta (t-{t}^{{\prime} })$. *τ*_p_ determines the timescale of change in ${\hat{{\bf{n}}}}_{i}$ ((cos(*θ*_*i*_), sin(*θ*_*i*_)) in two dimensions), the direction of the active force with strength *f*. *ζ* is the dissipative friction term, and **f**_**ij**_ = –∇*ϕ*({**r**_**ij**_}) are the interparticle forces. We integrate the equations of motion with a time step of d*t* = 0.01 following a BAOAB update scheme described elsewhere^47,48^. Details are included in the Supplementary Information.

### Stress-controlled cyclic shear simulations

Stress-controlled cyclic shear simulations are performed in the *NPT* ensemble in the LAMMPS package^46^ using the Parrinello–Rahman barostat^30,49^. The unit vectors describing the triclinic box are allowed to deform in response to the externally applied stress and the following equations of motion are used.

2 $$\begin{array}{rcl}{\dot{{\bf{x}}}}_{{\bf{i}}}&=&{{\bf{p}}}_{{\bf{i}}}/m+\gamma ({{\bf{x}}}_{{\bf{i}}}-{{\bf{R}}}_{{\bf{0}}})\\ {\dot{{\bf{p}}}}_{{\bf{i}}}&=&{F}_{i}-(\gamma +\nu ){{\bf{p}}}_{{\bf{i}}}\\ \dot{\nu }&=&\frac{1}{{\tau }_{{\rm{T}}}^{2}}\left(\frac{T(t)}{{T}_{0}}-1\right)-3\gamma \nu \\ \dot{s}&=&3(N-1)s\nu \\ \dot{\gamma }&=&\frac{V}{{\tau }_{{\rm{P}}}^{2}N{k}_{{\rm{B}}}{T}_{0}}\left({\bf{P(t)}}-{P}_{0}{\bf{I}}\right)+3\frac{{\tau }_{{\rm{T}}}^{2}}{{\tau }_{{\rm{P}}}^{2}}{\nu }^{2}{\bf{I}}\\ \dot{{\bf{L}}}&=&\gamma {\bf{L}}\end{array}$$

Here **L** = (**a**, **b**) is the matrix whose columns give the box length vectors, describing a parallelogram in two dimensions. *T*_0_ and *P*_0_ are the target temperature and pressure, respectively, whereas the corresponding instantaneous values are *T*(*t*) and *P*(*t*). *γ* represents the box tilt, leading to a deformed box with matrix **L**(**t**) whose area (or volume in three dimensions) is given by det(**L**(**t**)). Particle positions can be scaled as ${{\bf{x}}}_{{\bf{i(t)}}}={{\bf{L}}({\bf{t}}){{\bf{x}}}^{{\prime} }}_{{\bf{i}}}({\bf{t}})$, whereas distances are given by ${r}_{ij}^{2}(t)={{\bf{r}}}_{{\bf{ij}}}^{{\prime} }({\bf{t}}){{\bf{L}}}^{{\rm{T}}}({\bf{t}}){\bf{L}}({\bf{t}}){{\bf{r}}}_{{\bf{ij}}}^{{\prime} }({\bf{t}})$ (${{\bf{x}}}_{{\bf{i}}}^{{\prime} }({\bf{t}})$ are the positions in the box reference frame). *τ*_P_ and *τ*_T_ are timescales related to the spring constants of the thermostat and barostat, respectively, whereas *s* and *ν* are the corresponding auxiliary variables.

We perform stress-controlled cyclic shear simulations of 200 cycles, with a total of 16 independent samples (starting from different initial conditions at the given parent temperature).

The applied stress tensor **P**(**t**) has strictly zero diagonal entries and an off-diagonal shear stress *σ*_*xy*_(*t*), which is varied. We check to ensure that the box area fluctuations are negligible, as intended with the nature of the deforming stress applied. The stress is varied cyclically at a constant rate of $\dot{\sigma }=1{0}^{-3}{\tau }^{-1}$, which changes sign when the stress amplitude is reached (a sawtooth variation rather than sinusoidal). The strain rate is estimated by comparing raw displacements over a finite time window, yielding net velocities whose alignment with the shear direction $\hat{x}$ gives a measure of the strain rate. Data are averaged over different initial points and independent samples. The procedure discussed in the next section recovers the strain rate when applied to the case of stress-controlled cyclic shear.

### Strain, stress and strain rates resulting from active forcing

The alignment of velocities with the active force direction *v*_par_, averaged over independent configurations, produces a deformation field with which we identify a strain rate^18^.

3 $${v}_{{\rm{par}}}=\left\langle \mathop{\sum }\limits_{i=1}^{N}{{\bf{v}}}_{{\bf{i(t)}}}\cdot {{\bf{n}}}_{{\bf{i(t)}}}\right\rangle$$

In the case of stress-controlled cyclic shear, this expression can be rewritten as

4 $${v}_{{\rm{par}}}=\left\langle \mathop{\sum }\limits_{i=1}^{N}\frac{\Delta {{\bf{x}}}_{{\bf{i}}}({\bf{t}};\Delta {\bf{t}})}{\Delta t}\cdot \hat{x}\right\rangle,$$

where **x**_**i**(**t**)_ is the position vector and $\hat{x}$ is the direction of the deforming stress at any given point in time, leading to the following expression for the *x* coordinate in the deformed, raw coordinates: *x*(*t*) = *x*′(*t*) + *γ*(*y*(*t*) + *L*/2). Here *x*′(*t*) is the position in the box reference frame and *y*(*t*) is understood to be in a reference box centred at 0. For the case of active dynamics, following the approach in ref. ^16^, we consider the case of cyclic shear at a finite strain rate, where the affine component of the velocity of particle *i* is of the form $\dot{\gamma }{{\bf{r}}}_{i}\cdot \hat{y}$, and the magnitude of the resultant field is given by $\dot{\gamma }{[\sum {(\,{y}_{i})}^{2}]}^{1/2}$. Discounting the centre-of-mass motion and assuming a uniform distribution of particles along the *y* direction, one obtains a relationship of ${v}_{{\rm{par}}}^{{\rm{cs}}}=\dot{\gamma }{L}_{y}\sqrt{N/12}$. Retaining the pre-factor as an approximation for active dynamics, one can write the effective strain rate in active systems in terms of the deformation field as

5 $${\dot{\gamma }}_{{\rm{act}}}=\frac{\sqrt{12}}{\sqrt{N}L}{v}_{{\rm{par}}}^{{\rm{act}}}.$$

One can then define an instantaneous strain step, $\Delta \gamma ={\dot{\gamma }}_{{\rm{act}}}\Delta t$. The derivative of the potential energy with respect to the strain, $\scriptstyle\frac{{\rm{d}}E}{{\rm{d}}\gamma }$, yields the stress^16^ *σ*_act_, which we estimate from the parametric dependence of Δ*E* with respect to Δ*γ* over a given time step as *t*→*t* + Δ*t* (Supplementary Information):

6 $${\sigma }_{{\rm{act}}}=\frac{1}{{L}^{2}}\frac{{\rm{d}}E}{{\rm{d}}\gamma },$$

where *L* is the box length. We use a similar procedure in cyclically sheared simulations to compare the stress measured using this procedure, with that obtained directly from the virial stress tensor at the maximum strain *γ*_max_.

### Boundary interaction in confinement

We perform simulations of the same 65:35 Lennard–Jones mixture of *N* = 1,000 particles in elliptical confinement of constant shape and volume. Each particle interacts with the boundary wall with a truncated Lennard–Jones interaction where the wall-specific parameters are given by *ϵ*_w_ = 0.5*ϵ*_*αα*_, *σ*_w_ = 0.5*σ*_*αα*_ and *r*_cw_ = 3*σ*_AA_ (*α* denotes the particle type, either A or B).

The instantaneous distance of each particle from the confinement boundary is obtained by implementing an iterative procedure described elsewhere^50^ (see Supplementary Information for details). Heat maps showing the distance from an ellipse boundary calculated using this scheme are shown in the Supplementary Information. Visualizations of the particle assembly in confinement are prepared using the OVITO software suite^51^.

### Initial sample preparation in confinement

The initial configurations are prepared by first equilibrating a high-temperature liquid at *T* = 2.5. Uncorrelated configurations are then thermally annealed at different preparation temperatures *T*_p_ to obtain the samples in confinement (Supplementary Information shows the time evolution during thermal annealing). The annealed samples are then subjected to an instantaneous thermal quench to a very low temperature, *T* = 10^−4^, to approximate an energy minimization procedure.

### Cage-relative MSD

The cage-relative MSD^45^ is given by

7 $$\begin{array}{r}\Delta {{\bf{r}}}_{i,{\rm{CR}}}(t)=\Delta {{\bf{r}}}_{i}-\frac{1}{{N}_{{\rm{nn}}}}\mathop{\sum }\limits_{j=1}^{{N}_{{\rm{nn}}}}\Delta {{\bf{r}}}_{j}(t)\\\left\langle \parallel \Delta {r}_{{\rm{CR}}}(t){\parallel }^{2}\right\rangle =\left\langle \frac{1}{N}\mathop{\sum }\limits_{i=1}^{N}\parallel \Delta {{\bf{r}}}_{i,{\rm{CR}}}(t){\parallel }^{2}\right\rangle,\end{array}$$

where Δ**r**_*i*_(*t*) = **r**_*i*_(*t*) – **r**_*i*_(0), *N*_nn_ is the number of nearest neighbours within a shell of $1.5{\sigma }_{{\alpha }_{i}{\alpha }_{j}}$ from particle *i* at time *t* = 0. Further, to correct for motion due to global rotations without rearrangement, we identify the optimally rotated configuration {**R**(*θ*_min_)**r**_**i**_(*t*)}, where **R**(**θ**) is a matrix that performs a rotation by *θ* in two dimensions. The angle *θ*_min_ is determined by an optimization procedure that rotates each particle along a scaled ellipse of an identical aspect ratio as the confinement geometry (but with scaled axes):

8 $${\theta }_{{\rm{min}}}={\arg \min }_{\theta }\sqrt{\left\langle {\left[{{\bf{r}}}_{{\bf{i}}}({\bf{t}}+{\mathbf{\Delta }}{\bf{t}})-{\bf{R}}(\theta ){{\bf{r}}}_{{\bf{i}}}({\bf{t}})\right]}^{2}\right\rangle }.$$

The accumulated displacement vector between {**r**(*t* + Δ*t*)} and the optimally rotated {**R**(*θ*_min_)**r**_**i**_(*t*)} is tracked and its magnitude gives the instantaneous cage-relative MSD.

9 $$\begin{array}{rcl}\Delta {{\bf{r}}}_{i,{\rm{CR}}}(t)&=&\Delta {{\bf{r}}}_{i,{\rm{CR}}}(t-\Delta t)+\Delta {{\bf{r}}}_{i}^{{\theta }_{{\rm{min}}}}(t)-\frac{1}{{N}_{{\rm{nn}}}}\mathop{\sum }\limits_{j=1}^{{N}_{{\rm{nn}}}}\Delta {{\bf{r}}}_{j}{(t)}^{{\theta }_{{\rm{min}}}}\\ \Delta {{\bf{r}}}_{i}^{{\theta }_{{\rm{min}}}}(t)&=&{{\bf{r}}}_{{\bf{i}}}(t)-{\bf{R}}({\theta }_{{\rm{min}}}){{\bf{r}}}_{{\bf{i}}}(t-\Delta t).\end{array}$$

Note that although the initial centre-of-mass force and angular momentum are set to zero by appropriately adjusting the active force orientation vectors, the subsequent free diffusion of the orientation vectors does not prevent global motion.

### Curvature and alignment definitions for confined active dynamics

The curvature of an ellipse is parametrically defined using the following equation:

10 $$\kappa =\frac{ab}{{\left(\sqrt{{a}^{2}{\sin }^{2}(\theta )+{b}^{2}{\cos }^{2}(\theta )}\right)}^{3}},$$

where *a* and *b* are the major- and minor-axis lengths, respectively, of the confinement geometry and *θ* is the parametric angle for a point on the boundary. We consider the particles whose centres are within *σ*_AA_ of the wall and characterize their motion with respect to their neighbours over finite time windows.

A particle of interest *i* moves along a vector **r**_**i**_(**t** + Δ**t**) – **r**_**i**_(**t**) over a window Δ*t*. The angle that this vector makes with respect to the *x* axis, *ω*_*i*_, is similar to the angles *ω*_*j*_ of neighbours of *i*, labelled *j*, if the displacement vector(s), **r**_**j**_(**t** + Δ**t**) – **r**_**j**_(**t**), are similarly oriented. We, thus, compute the difference Δ*ω*_*ij*_(*t*; *t* + Δ*t*) and average over particles *i* near the boundary, and their neighbours *j* (within $2.5{\sigma }_{{\alpha }_{i}{\alpha }_{j}}$ (*α*_*i*_ is the type of particle *i*)), over multiple snapshots separated by 10^3^*τ* and eight independent simulations. The parametric dependence of the cosine of Δ*ω*_*ij*_ with the curvature *κ* is investigated (Supplementary Fig. 22).

## Online content

Any methods, additional references, Nature Portfolio reporting summaries, source data, extended data, supplementary information, acknowledgements, peer review information; details of author contributions and competing interests; and statements of data and code availability are available at 10.1038/s41567-025-02843-7.

## Supplementary information

Supplementary Information Supplementary Sections 1–13 and Figs. 1–22.

Supplementary Video 1 Time lapse of the trajectory for *N* = 1,000 particles simulated in confinement in an ellipsoid of *e* = 1.2, with *f* = 0.6, showing large-scale rearrangements involving coherent motion.

Supplementary Video 2 Time lapse of the trajectory for *N* = 1,000 particles simulated in confinement in an ellipsoid of *e* = 1.8, with *f* = 0.6, showing relative quiescence with vibrations of particles around their mean positions.

## Data Availability

The dataset for this study is available via Figshare at 10.6084/m9.figshare.28189739.

## Code availability

Scripts and custom-written codes are available via GitLab at https://gitlab.com/sastry_lab/codes/activeyieldingconfinement.
