# Supplementary material for: Yielding behaviour of active particles in bulk and in confinement
Source: Nat Phys. 2025 Mar 31;21(5):817–24. doi: 10.1038/s41567-025-02843-7 (PMC12084156; doi:10.1038/s41567-025-02843-7)
Supplement: Supplementary file 1 — Supplementary Sections 1–13 and Figs. 1–22. [file 41567_2025_2843_MOESM1_ESM.pdf]

nature physics

Supplementary information https://doi.org/10.1038/s41567-025-02843-7

Yielding behaviour of active particles in bulk and in confinement

In the format provided by the authors and unedited

Contents

S1. Integration of equations of motions for active dynamics 2

S2. Sample preparation 3

S3. Yielding phase diagram at different $\tau_{\mathbf{p}}$ 4

S4. Parameteric dependence of change in potential energy, ΔE, on strain step $\Delta\gamma$ 4

S5. Exponents governing time to steady state, strain rate, and flow 5

S6. Calculating stress for cyclic shear from the parametric dependence of ΔE on $\Delta\gamma$ 8

S7. Mean squared displacements close to yield 9

S8. Comparison of timescale to steady state from stretched exponential fits and from first passage time identification 12

S9. Comparison with passive dynamics 13

S10. Fluidisation induced by passive thermal dynamics 15

S11. Energy relaxation and timescale to steady state at different τ 16

S12. Confinement simulations 19

A. Confinement boundary implementation 19

B. Sample preparation in confinement 20

C. Time evolution of energy in confinement 20

D. Particle density correlation and maps in confinement 22

S13. Description of Supplementary Videos 24

References 25

S1. Integration of equations of motions for active dynamics

The discretized time integration is done following the prescription in [1, 2] for the **BAOAB** operator splitting to include the active force along the orientation vector, $\hat{n} = (cos(\theta), sin(\theta))$.

Given the equations of motion:

$$\begin{aligned}
\dot{\mathbf{p}}_i &= -\zeta \mathbf{p}_i + \sum_{j \neq i=1}^{N} (\mathbf{f_{ij}}) + f\hat{\mathbf{n}}_i + \xi_{\mathbf{t}}{}^i \\
\dot{\mathbf{x}}_i &= \mathbf{v}_i \equiv M^{-1}\mathbf{p}_i \\
\dot{\theta}_i &= \xi_{\mathbf{r}}{}^i
\end{aligned} \tag{S1}$$

we write

$$\mathrm{d}\begin{bmatrix}\mathbf{x}\\ \mathbf{p}\\ \theta\end{bmatrix} = \underbrace{\begin{bmatrix}M^{-1}\mathbf{p}\\ 0\\ 0\end{bmatrix}\mathrm{d}t}_{\mathrm{A}} + \underbrace{\begin{bmatrix}0\\ -\nabla\mathbf{U} + f\hat{\mathbf{n}}\\ 0\end{bmatrix}\mathrm{d}t}_{\mathrm{B}} + \underbrace{\begin{bmatrix}0\\ -\zeta\mathbf{p}\mathrm{d}t + \sqrt{2\zeta M k_B T}\mathrm{d}\mathbf{w}\\ \sqrt{2/\tau_p}\mathrm{d}\mathbf{w}\end{bmatrix}}_{\mathrm{O}}, \tag{S2}$$

The stochastic component is modelled on the Wiener process $\mathbf{w}$, which follows:

$$\mathrm{d}\mathbf{w} = \mathbf{w}(t + \mathrm{d}t) - w(t) = \sqrt{\mathrm{d}t}\mathbf{GR}(\mathbf{0}, \mathbf{1}; \mathbf{t}) \tag{S3}$$

where $\mathbf{GR}(\mathbf{0}, \mathbf{1}; \mathbf{t})$ is a vector of independent, Gaussian distributed random variables with means 0 and variance 1. For the active particle system, one needs to include the active forces in the velocity update of the **BAOAB** integrator, and as well perform the time integration for the orientation, $\theta(t)$.

Defining constants,

$$c_1 = e^{-\zeta\delta t}, \quad c_2 = \zeta^{-1}(1 - c_1),$$

$$c_3 = \sqrt{k_B T(1 - c_1^2)}, \quad c_4 = \sqrt{2\ dt/\tau_p},$$

we have the update steps:

$$\mathbf{p}_i\left(t+\frac{\mathrm{d}t}{2}\right)=\mathbf{p}_i(t)+\frac{\mathrm{d}t}{2}\left[-\nabla\mathbf{U}(\mathbf{x}_i(t))+f\hat{\mathbf{n}}_\mathbf{i}(\mathbf{t})\right]; \tag{S4a}$$

$$\mathbf{x}_i\left(t+\frac{\mathrm{d}t}{2}\right)=\mathbf{x}_i(t)+\frac{\mathrm{d}t}{2}\mathbf{M}^{-\mathbf{1}}\mathbf{p}_i\left(t+\frac{\mathrm{d}t}{2}\right); \tag{S4b}$$

$$\hat{\mathbf{p}}_i\left(t+\frac{\mathrm{d}t}{2}\right)=c_1\mathbf{p}_i\left(t+\frac{\mathrm{d}t}{2}\right)+c_3\mathbf{M}^{\mathbf{1/2}}\mathbf{GR}_i(0,1;t+dt); \tag{S4c}$$

$$\theta_i(t+dt)=\theta_i(t)+c_4\mathbf{GR}_i^{'}(0,1;t+dt); \tag{S4d}$$

$$\mathbf{x}_i(t+\mathrm{d}t)=\mathbf{x}_i\left(t+\frac{\mathrm{d}t}{2}\right)+\frac{\mathrm{d}t}{2}\mathbf{M}^{-\mathbf{1}}\hat{\mathbf{p}}_i\left(t+\frac{\mathrm{d}t}{2}\right); \tag{S4e}$$

$$\mathbf{p}_i(t+\mathrm{d}t)=\hat{\mathbf{p}}_i\left(t+\frac{\mathrm{d}t}{2}\right)+\frac{\mathrm{d}t}{2}\left[-\nabla\mathbf{U}(\mathbf{x}_i(t+\mathrm{d}t))+f\hat{\mathbf{n}}_i(t+\mathrm{d}t)\right]. \tag{S4f}$$

(S4g)

S2. Sample preparation

We show the inherent structure potential energies, E_{IS}, as a function of time as well as the dependence of the steady state inherent structure energies, E_{IS}, as a function of the temperature at which the simulation is performed, T_p, in Fig. S1.

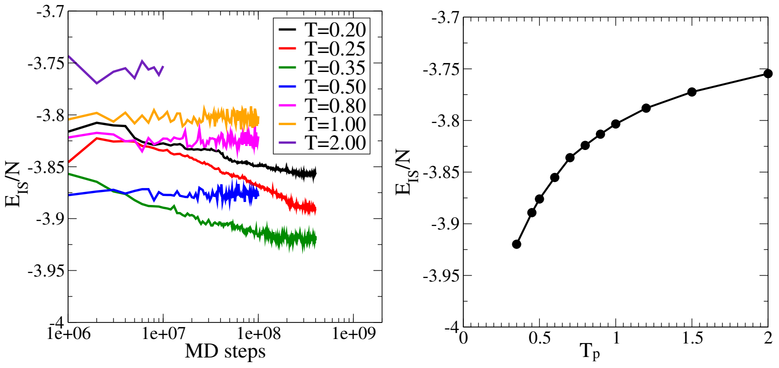

FIG. S1. Average inherent structure energy per particle as a function of time for $N = 1000$ particles of the binary Lennard-Jones mixture in 2D, simulated in the NVT ensemble at different temperatures. The steady state average inherent structure energy per particle, E_{IS}, is shown as a function of simulation temperature for the cases at which a steady state is reached, i.e., $T \geq 0.45$.

S3. Yielding phase diagram at different τ_p

In Fig. S2 we show the full yielding phase diagram with active dynamics at $\tau_p = 1.01 \times 10^4 \tau$ and $\tau_p = 1.01 \times 10^5 \tau$.

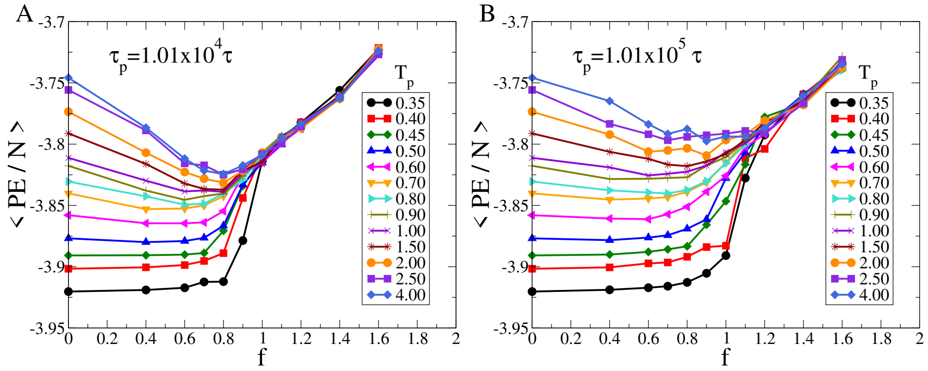

FIG. S2. Yielding phase diagram for $N = 1000$ particles of the $65 : 35$ binary Lennard-Jones mixture in 2D subjected to active dynamics at $\tau_p = 1.01 \times 10^4 \tau$ and $\tau_p = 1.01 \times 10^5 \tau$ (right).

S4. Parameteric dependence of change in potential energy, ΔE, on strain step $\Delta\gamma$

The alignment of instantaneous particle velocities with the respective directions of active forcing yield a measure of the instantaneous strain rate as in Eq. S5 below [3]. In systems driven by active forces or using a shear stress, the strain rate response changes drastically across the yield point [3, 4]. We define the instantaneous strain rate from the alignment of velocities with the active force direction as

$$v_{par}^{act} = \left\langle \sum_{i=1}^{N} \mathbf{v_i(t)} \cdot \mathbf{n_i(t)} \right\rangle_t$$
$$\dot{\gamma}_{act} = \frac{\sqrt{12}}{\sqrt{N}L} v_{par}^{act}. \tag{S5}$$

From the instantaneous strain rate, $\dot{\gamma}_{act}(t)$, we define an instantaneous step $\Delta\gamma = \dot{\gamma}_{act}\Delta t$ and consider the change in potential energy with respect to it. We assume a linear parametric dependence of ΔE on $\Delta\gamma$ of the form $y = \beta x + \xi$, where ξ is a noise term, and identify the slope β, taken as the Pearson correlation times the ratio of the standard deviations, $\sigma_{\Delta\gamma}/\sigma_{\Delta E}$. As mentioned in the main text, this is an approximate procedure that provides estimates of the stress using the response of the change in potential energy to changes in

the measured strain step. As shown in Fig. 1 (d) of the main manuscript, and discussed below, such an approximation is found to give estimates in reasonable agreement close to the maximal strain γ_{max} when used in the context of cyclic shear simulations at finite rate.

S5. Exponents governing time to steady state, strain rate, and flow

Yielding in actively driven systems and in stress-controlled cyclic shear simulations has been shown to be characterised by the development of a non-zero strain-rate beyond yield. This is also related to a diverging timescale to reach steady state at the yield point, which we discuss in Fig. 2 of the main manuscript. Here, we discuss further the scaling exponents for the divergence of time scales near the yield point, and the flow curves after yielding, for which the following relationships have been discussed in literature [5]:

$$\tau_f = \frac{A}{\dot{\gamma}^{\alpha}} \tag{S6}$$

$$\tau_f = \frac{B}{(\sigma - \sigma_{yield})^{\beta}} \tag{S7}$$

$$\dot{\gamma} = \left(\frac{(\sigma - \sigma_{yield})}{K}\right)^{1/n} \tag{S8}$$

where τ_f is the fluidization time, $\dot{\gamma}$ is the strain rate, σ_{yield} is the yield value of the stress σ (equivalently f_{yield} and f for active driving). The Herschel-Bulkeley exponent, n, is thus related to the exponent α relating the non-zero strain rate and the fluidization time beyond yield, and exponent β that relates the fluidization time to the distance to the yield point. There is a wide range of exponent values that have been reported, even when we restrict attention to a single phenomenon and protocol (*e. g.*, uniform shear at finite shear rate of a soft solid).

The factors contributing to such variability has been discussed [5], and may include sample preparation methods, boundary conditions, [6–8], packing fraction[9], strength of the mechanical coupling between different parts of a material [8], system size effects and indirect estimates through scaling arguments [10], to list a few.

Active Dynamics: In Fig. S3, we summarise the results from investigating the behaviour of the time to reach the steady state and the strain rate with changing active force. For active dynamics starting from well-annealed initial states, the time evolution of the potential

energy is used to obtain t_{ss} (see Fig. 2 (C,D) in the main manuscript), and the resultant data fit to a power law to obtain $f_{yield} = 0.699$ and $\beta = 3.984$. In Fig. S3, we summarise the results from investigating the behaviour of the time to reach the steady state, t_{ss} (τ_f in Eq. S8), and the strain rate, $\dot{\gamma}$, with changing active force. We obtain values for the exponents, $\alpha = 2.906$ and $n = 0.813$, noting that data at large $\dot{\gamma}$ have been described in the literature to deviate from the behaviour exhibited close to the yielding transition [11]. We thus only show data in Fig. S3 (A) and (B) for $\dot{\gamma}$ and f close to yield, where a clear scaling relation can be identified. A comparison of our subsequent results with shear driving, as

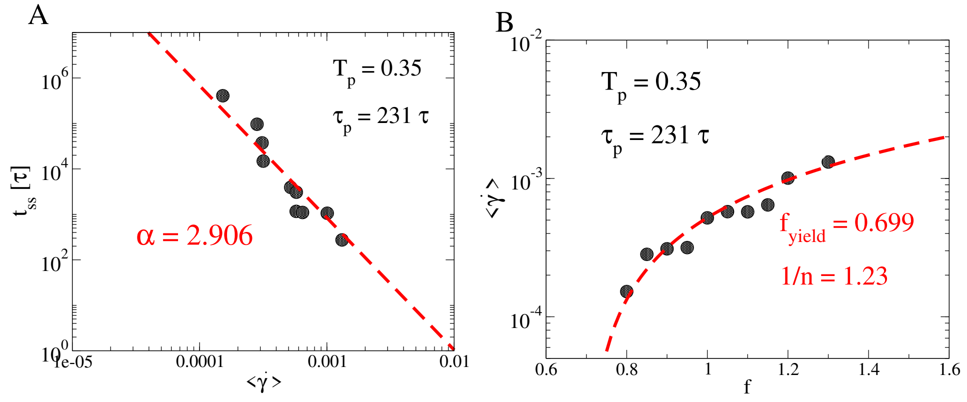

FIG. S3. In panel (A), we show the behaviour of t_{ss} against $\dot{\gamma}$, which tends to 0 as the timescale diverges, obtaining a value of $\alpha = 2.906$ (see Eq. S8). In panel (B) we fit the dependence of the strain rate on $f - f_{yield}$ to a power law form as in Eq. S8 with an exponent of $n = 0.813$. f_{yield} is set as a fixed parameter, obtained from the value of f at which the extrapolated t_{ss} is expected to diverge ($f = 0.699$), based on fits to the data in Fig. 2 (D) in the main manuscript. The relation, $n = \alpha/\beta$ leads to a similar expected value of 0.729, where $\beta = 3.984$ (see Fig. 2 (D) of the main manuscript).

well as those in literature for shear driven and active systems, reveals that two dimensional systems typically exhibit Herschel-Bulkeley exponents n in the range of $0.75 - 0.85$, which are considerably larger than the mean field value of $1/2$.

Stress-controlled cyclic shear: We consider yielding in cyclically sheared systems where the shear stress is controlled, and investigate the timescales and developing strain rate beyond the yield point, σ_{yield}, and summarise these results in Fig. S4.

The time evolution of the potential energy and the behaviour of t_{ss} as a function of σ_{xy}^{max} is shown in Fig. 2 (E,F) of the main manuscript, where the estimated yield stress is obtained as $\underline{\sigma_{yield} = 0.735}$ and the exponent, $\underline{\beta = 1.87}$. The parametric dependence of t_{ss} on the measured strain rate, $\dot{\gamma}$, is shown in Fig. S4 (A) yielding $\alpha = 1.51$. Finally, the strain rate as a function of maximal shear stress is shown in Fig. S4 (B), and fit to the Herschel-Bulkeley form with $\sigma_{yield} = 0.735$ (obtained from the extrapolated divergence of t_{ss}) to give an exponent, $n = 0.806$, which is consistent with the relation $n = \alpha/\beta = 0.807$ and with some corresponding results in the literature [4, 10]. However, we reiterate that the exponent values reported in the literature have a wide range, and note that our results result from those in [3, 12, 13].

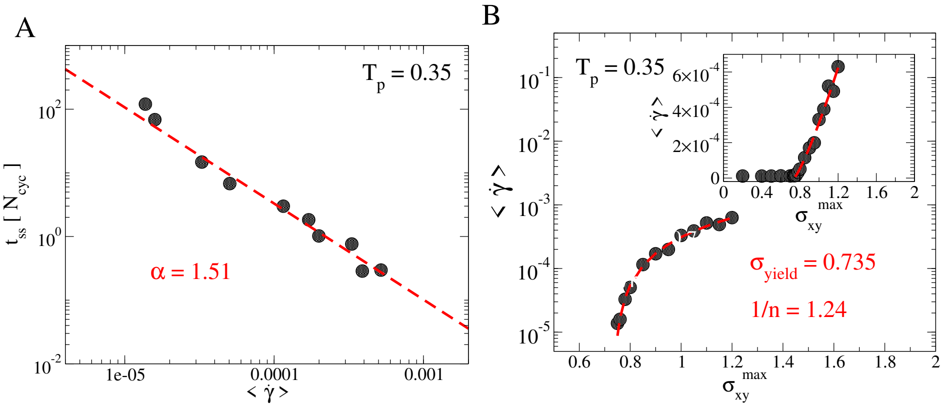

FIG. S4. In panel (A) we investigate the dependence of t_{ss} and $\dot{\gamma}$, with an α exponent of $\underline{2.906}$. In panel (B), we show the $\dot{\gamma}$ developing beyond yield, with $\sigma_{yield} = 0.735$ set as a fitting parameter from the extrapolated divergence of t_{ss} in Fig. 2 (F) in the main manuscript, yielding a Herschel-Bulkeley exponent of $\underline{n = 0.806}$.

Uniform shear at fixed shear rates: In order to make a comparison with exponents in a strain controlled protocol for our system, we perform uniform shear simulations at a finite shear rate and temperature, propagating the SLLOD equations of motion using the LAMMPS software suite[14]. Simulations are performed with a time step of $dt = 0.01$, $T = 10^{-3}$, at different strain rates, $\dot{\gamma}$.

The results are shown in Fig. S5, with the flow curves for different strain rates shown in Fig. S5 (A) and the average steady state flow stress shown as a function of the strain rate in Fig. S5 (B). The Herschel-Bulkeley exponent and the yield stress are independently esti-

mated, giving a $\sigma_{yield} = 0.723$ and an HB exponent of $n = 0.853$, broadly agreeing with the estimates from stress-controlled cyclic shear.

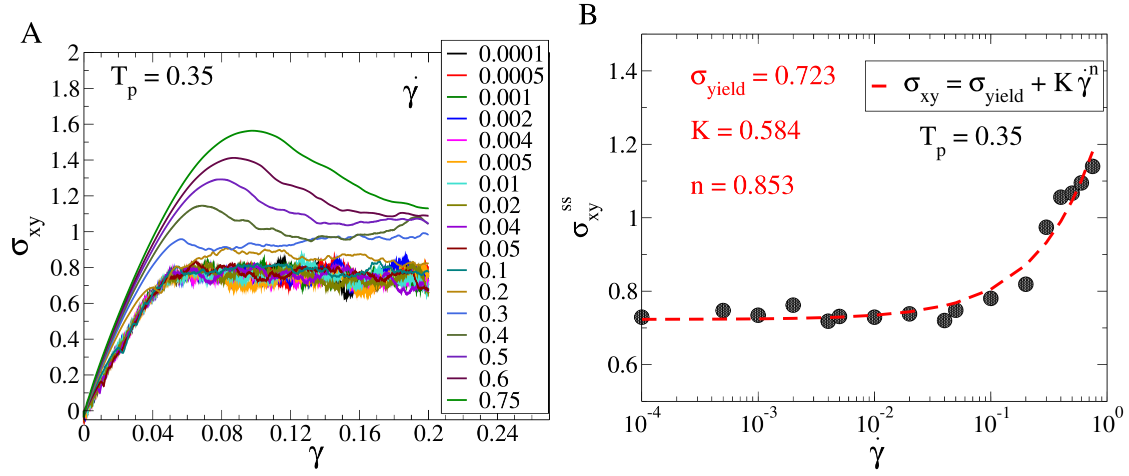

FIG. S5. Stress vs strain from uniform shear of well-annealed samples (averaged over 16 independent runs) at different applied strain rates in panel (A). The steady state shear stress beyond yield is estimated and shown as a function of the applied strain rate, with the corresponding data fit to the Herschel-Bulkeley form to extract the exponent, n.

S6. Calculating stress for cyclic shear from the parametric dependence of ΔE on $\Delta\gamma$

In Fig. 1 (d) of the main text, we show the dependence of the maximum stress on γ_{max} in the case of cyclic shear, where the stress is computed by finding the slope of the best linear fit to the scatter of ΔE vs $\Delta\gamma$ from cyclic shear simulations at constant shear rate. The velocity stress component, $\sum_{i=1}^{N} \delta v_x^i \delta v_y^i$ (the δ represents change with respect to any global flow), is subsequently added. This stress value is compared to that obtained directly from the virial stress tensor for γ near γ_{max}. More specifically, we first consider a reference γ close to γ_{max} ($\gamma_{max} - 10^{-4}$ and note the energy E_{ref}. We then consider the energy of configurations within a neighbourhood of $\Delta\gamma = 10^{-4}$ of γ_{ref} (identified from the strain rate multiplied with time windows Δt) and store the corresponding $\Delta\gamma$ and $\Delta E = E - E_{ref}$. The parametric dependence of ΔE and $\Delta\gamma$ is used as in active systems to determine the slope. Such a procedure is limited in this case to conditions where the strain step is very small, such that plastic rearrangements and other non-affine motion does not obscure the dependence of ΔE on the strain step. The data of ΔE vs $\Delta\gamma$ is aggregated over 10 steady state cycles from 16

independent simulations. In Fig. S6, we show a comparison of the Virial shear stress with the estimated shear stress from the procedure described here.

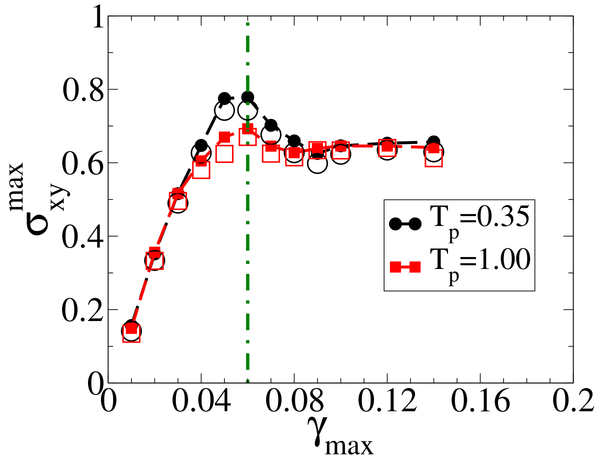

FIG. S6. The Virial shear stress (solid symbols) compared with the shear stress obtained from the parameteric dependence of ΔE on $\Delta\gamma$ for well-annealed and poorly annealed samples, averaged over configurations in the steady state close to γ_{max}, and from 16 independent simulations.

S7. Mean squared displacements close to yield

In Fig. S7, we show mean squared displacements at one value of the persistence time, $\tau_p = 231$, for multiple values of f close to the window where yielding is expected, based on the divergence of t_{ss} in Fig. 2 (D) in the main manuscript. We also show the mean squared displacements for poorly annealed samples in Fig. S7 (A). Straight line fits to the logarithm of the mean squared displacements against the logarithm of the time window are used to extract the diffusivities reported in Fig. 2 (B) of the main manuscript, wherever such a straight line fit of unit slope describes the data meaningfully on the log-log scale. For $f < 0.7$, clearly the mean squared displacements are sub-diffusive with very small average particle displacements of less than 0.1 σ_{AA} over time windows of around 10^6 τ. Since the mean squared displacements for small diffusivities exhibit a plateau preceding the diffusive regime, the duration of runs must be long enough for the diffusive displacements to exceed the cage size. Thus, we need $2dDt > < r^2_{cage} >$ where $< r^2_{cage} >$ is the mean squared displacement at times for which the dynamics is non-diffusive. We find $< r^2_{cage} >$ to be less than $0.01\sigma^2_{AA}$ for $f < 0.7$ over timescales less than 10^5 τ, for the f values of interest (and

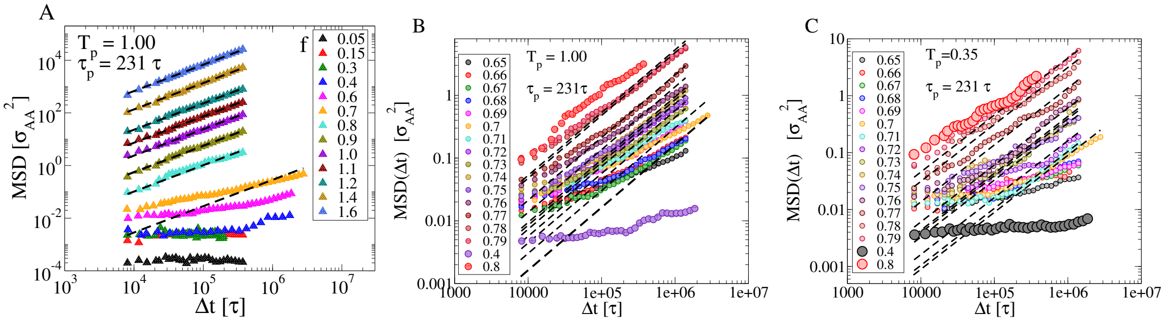

FIG. S7. Panel (A) Mean squared displacements for poorly annealed samples subjected to active dynamics at $\tau_p = 231\tau$, with results shown for more values of f close to the yield point in panel (B). Panel (C) shows the mean squared displacements for well-annealed samples, with the same range in f as in panel (B). Dashed lines in each case are straight line fits of unit slope for the logarithm of the MSD as a function of the logarithm of the time window, Δt.

lower, for smaller f). We compute $< r^2_{cage} >$ for simulation time 4×10^3. Considering the fit to the diffusivities, and the $< r^2_{cage} >$ as a function of f, as shown in Fig. S8, we can estimate the minimum run lengths as

$$t_{min} = \frac{\langle r_{cage} \rangle^2}{2dD}. \tag{S9}$$

The estimated run lengths are shown in Fig. S8 (C), for which a power law fit to the diffusivities, with a vanishing of the diffusivity at around $f = 0.43$, and a fit to $< r^2_{cage} >$ as being proportional to the effective temperature (including a $f^2\tau_p$ contribution), are used. Also shown are the run lengths used (run lengths of 6×10^8 MD steps or times $6 \times 10^6 \tau$ were performed for several f values in the range $0.6 \leq f \leq 0.8$), which have been chosen so that the extrapolated diffusivities should be accessible for $f \geq 0.6$.

As a further check, following [15], we consider log-log plots of the diffusivity *vs.* $f - f_{yield}$ where several choices are considered for f_{yield}, around the values we estimate from the drop in the diffusivity ($f_{yield} \approx 0.7$), as well as the divergence of times to the steady state ($f_{yield} = 0.7$ for $T_p = 0.35$ and $f_{yield} = 0.67$ for $T_p = 1.0$). The log-log plot of diffusivity *vs.* f in Fig. S9 shows a flattening at small f, with a linear relationship in the yielded regime, indicating that the diffusivity attains a finite value, rather than vanishing with a power law

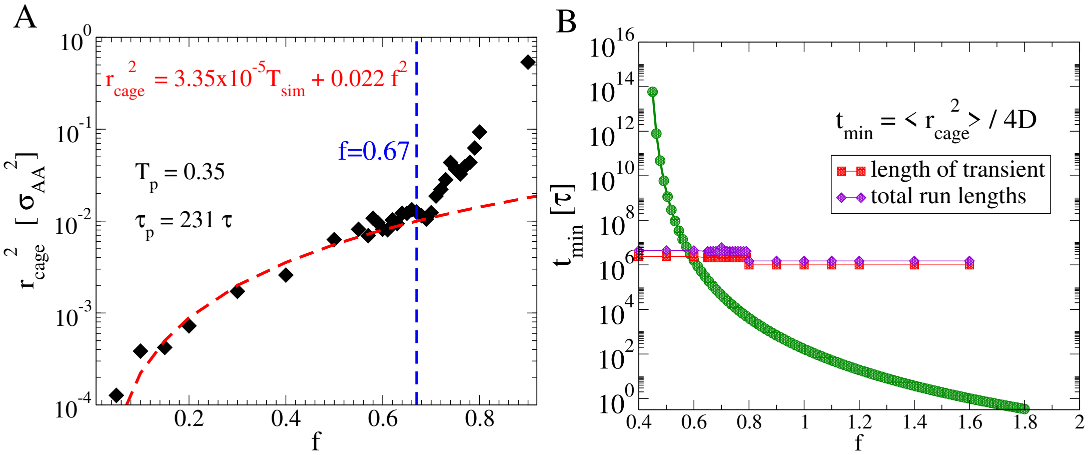

FIG. S8. The extrapolated diffusivity from Fig. 2 (B) in the main manuscript is used to estimate the minimum expected waiting time for particles to exit the cage of their local neighbours. In panel (A), we show the caging lengthscale as a function of f with the respective fit shown as a red-dashed line. Panel (B) shows the waiting time extrapolations, obtained from the relationship in Eq. S9 between the respective fits. The purple data points mark the total run lengths (for sets of runs at both sample preparation temperatures) at the corresponding f values, while the red data points mark the lengths of the minimum transients that are omitted at each f before configurations, presumed to be in the steady state, are used to compute the MSD.

form, at f_{yield}.

We believe that these additional results convincingly address the reviewer's concern regarding the discontinuous change of diffusivity at the fluidization transition.

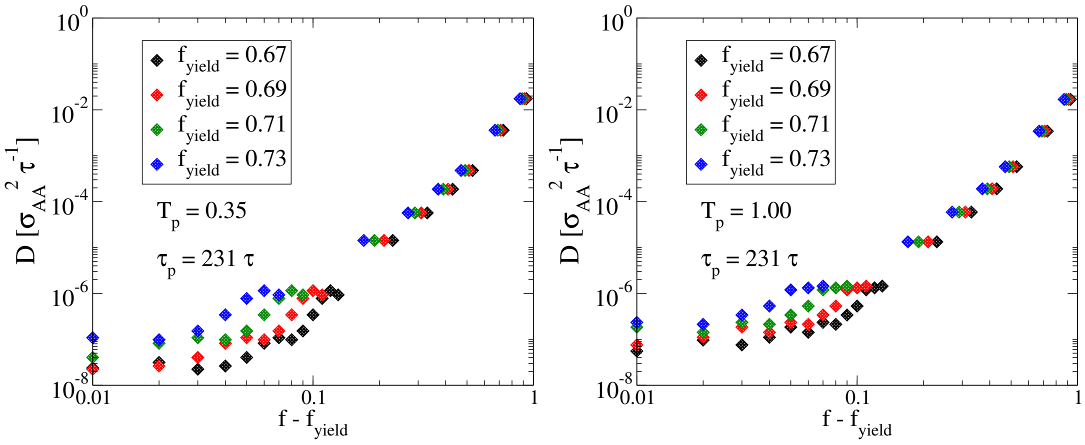

FIG. S9. The diffusivity plotted vs $f - f_{yield}$ for different candidate f_{yield}, each less than that measured from the timescale divergence in Fig. S3.

S8. Comparison of timescale to steady state from stretched exponential fits and from first passage time identification

In Fig. S10, we show the average time to reach state obtained from two different procedures. In the first we consider the time series, $\langle E \rangle$ vs time, averaged over 8 independent simulations and fitted a stretched exponential to the curve, to identify the relaxation timescale t_{ss} from Eq. S10. Next, we consider the average first passage time to the steady state energy $< E^i_{ss} >_{t_{ss}}$ in each trajectory i. Here, the average is taken over a window of time t_{ss} where the system is in its final steady state. The average of these first passage times is then taken over 8 independent simulations.

We note that for the poorly annealed case, trajectories undergo further slow annealing at small active forces, reminiscent of creep, after they enter into the neighbourhood of its final steady state. Likewise, for trajectories close to the transition point, the approach to steady state is extremely slow. This leads to the appearance of a shorter first passage time to steady state than that obtained from estimates of the relaxation timescale in a stretched exponential process, as seen in Fig. S10. While this may be ameliorated by improved estimates of the steady state energy, $\langle E^i_{ss} \rangle_{t_{ss}}$, reached by each trajectory, we opt for the stretched exponential procedure in our work in order to identify an asymptotic steady state energy. Trajectories subjected to active force significantly larger than the critical force yield similar estimates for $\langle t_{ss} \rangle$ from these two approaches.

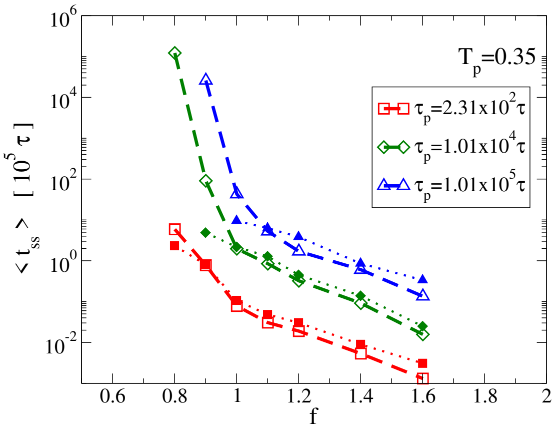

FIG. S10. Timescale to steady state shown at different values of τ_p for well-annealed samples at $T_p = 0.35$ subjected to active dynamics at forces greater than the yielding force. The hollow symbols with dashed lines are obtained from stretched exponential fits to the aggregate $\langle E \rangle$ vs time curves where the energy at each time point is the average over 8 independent simulations. The solid symbols with dotted lines are obtained from identifying the average of the first passage time to the steady-state value of the potential energy E_{ss} in each trajectory, where the average is over 8 independent simulations.

S9. Comparison with passive dynamics

We address the question of whether purely passive dynamics at comparable temperatures can lead to fluidisation in the same way that active dynamics does. In order to do this, we consider the average kinetic energy per particle across different cases. In Fig. S11, we show the average potential energy per particle for active dynamics where the active direction re-orients every timestep, dt, which we use to represent the limit of zero persistence where the direction of active force is delta-correlated in time. In this limit of small persistence, the active temperature is given by the expression $T_a = f^2\tau_p/4$ in 2D and the effective temperature is given by $T_{eff} = T_{bath} + T_a$. This relation holds exactly in the small persistence time limit, as we see in Fig. S11. We reiterate here that the kinetic energies at finite persistence are considerably lower than that for the small persistence time case. One does not, however, expect the same expression for T_a to hold at large τ_p. It has been argued in the literature that T_a goes as $Af^2\tau_p/(1 + B\tau_p)$[16, 17], but results reported in [17, 18] suggest that for the large persistence times we consider in the present manuscript, the above

expression may not be valid, with the arrest line exhibiting non-monotonic dependence on f.

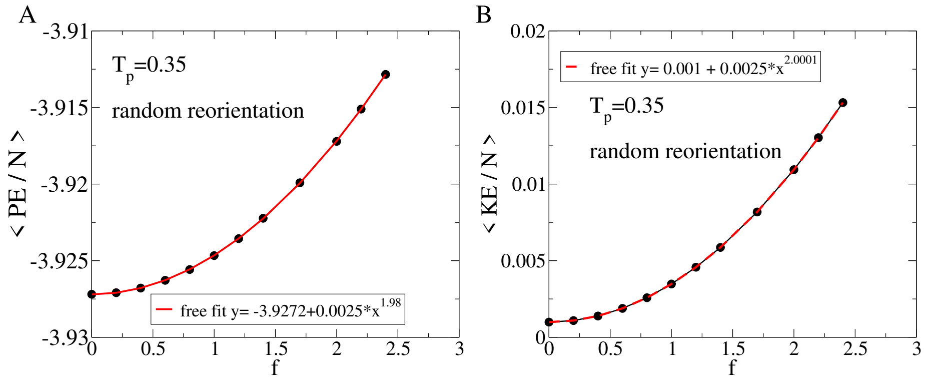

FIG. S11. No discontinuity in energy for zero persistence active force with a quadratic dependence of potential energy indicative of increased thermal motion. Quadratic dependence of kinetic energy on active force.

In Fig. S12 we show the average kinetic energy for simulations at different active force at finite and small τ_p. The average kinetic energy per particle remains low in these cases, and this is underscored by the fact that it remains below the kinetic energy for the small persistence case, where the kinetic energy can be compared with an exact expression of the effective temperature. The average kinetic energy per particle is higher in this low persistence case compared to that at finite persistence time.

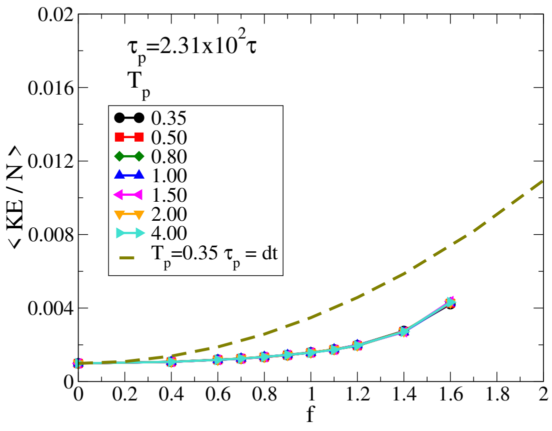

FIG. S12. Effective temperature from the kinetic energy per particle for differently annealed samples subjected to active forces at either finite persistence time, or at zero persistence for $T_p = 0.35$.

S10. Fluidisation induced by passive thermal dynamics

We consider passive dynamics, beginning from the differently annealed initial inherent structures, over a range of bath temperatures, T_{sim}, across the transition from the arrested to the diffusive state. These results show that for a "melting" transition of this kind, there is no real annealing dependence, nor a divergence in the relaxation time to the steady state as in the case of cyclic shear yielding or active yielding. The inherent structure energies in

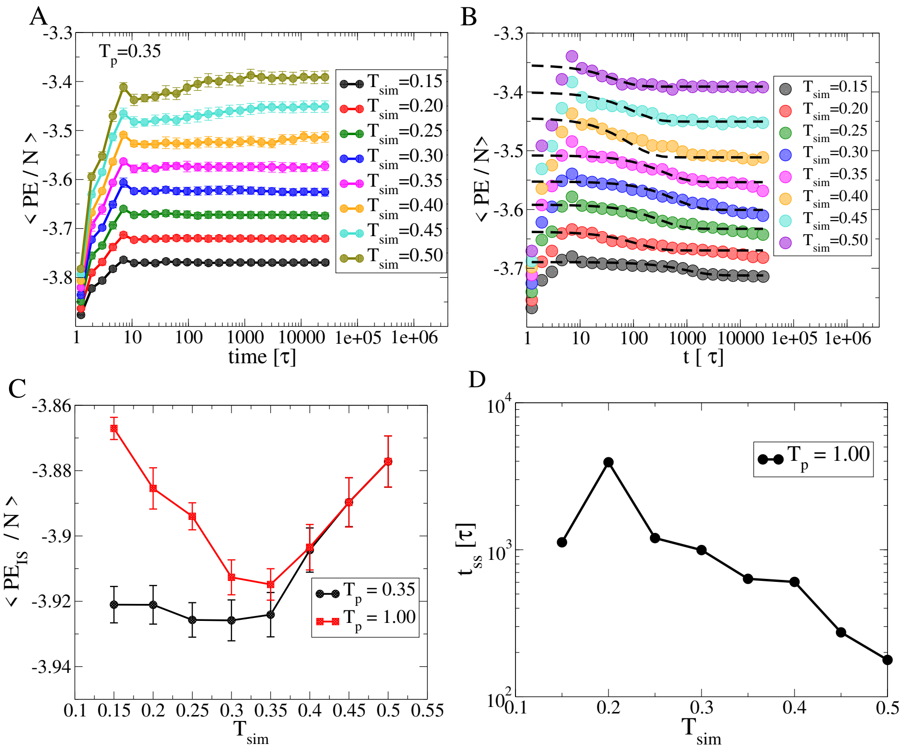

FIG. S13. Potential energy over time for a well-annealed sample (A) and a poorly annealed sample (B), when simulated at different bath temperatures T_{sim} using passive NVT dynamics. The black dashed lines in panel (B) are stretched exponential fits of the form in Eq. S10. Error bars in (A) and (B) denote standard deviations. Panel (C) shows the inherent structure potential energies, obtained by performing conjugate gradient energy minimisations of configurations. Data shown are obtained by averaging over 8 independent trajectories. Error bars denote standard deviations at the respective T_{sim}. Panel (D) shows the time to steady state for the poorly annealed case, which shows an overall increasing trend with no clear indication of a divergence near the transition.

Fig. S13 show signatures of a transition from annealing (or an absorbing steady state for well-annealed samples) to yielding across $T_{sim} = 0.30$. The timescale to steady state, here

measured for the poorly annealed sample by fitting the regime of relaxation to a stretched exponential form in Fig. S13, does not show any evidence of diverging across this range of temperatures, instead showing an overall slowing down of dynamics at progressively lower T_{sim}, as seen in Fig. S13 (D).

S11. Energy relaxation and timescale to steady state at different τ

We show the time series of average potential energy per particle over time for 8 independent trajectories simulated with different values of the active force. The energy vs time curves are fit to stretched exponential fits as shown in Fig. S14 for configurations prepared at $T_p = 0.35$ and in Fig. S15 for configurations prepared at $T_p = 1.00$. The fitting function has the following form:

$$\langle E(t) \rangle = E_0 + \Delta E \exp(-(t/t_{ss})^{\beta_{stretch}}), \tag{S10}$$

controlling for E_0 and ΔE based on the bounds in the trajectory. In each case, in the post-yield regime, the region used for the fit includes the time interval immediately preceding the sharp change that signals the transition and the steady state reached after the transition (see for example the case of $T_p = 1.00$, $\tau_p = 1.01 \times 10^5 \tau$ and $f \geq 1.2$, where the system undergoes an initial relaxation before transitioning to the fluidised state). Simulations performed with f values close to the transition point ($f = 0.8$ in Fig. S14 (A), $f = 0.9$ in Fig. S14 (B) and $f = 1.0$ in Fig. S14 (C)) reach the steady state very slowly, as a result of which the observed final values of $\langle PE/N \rangle$, reported in Fig. 3 of the main manuscript, are apparently intermediate to the values in the pre-yield and the post-yield branches. For f values below the critical value, it is difficult to define a time scale to reach the steady state for well annealed samples, since the system does not exhibit significant structural relaxation. Hence, we do not discuss the time to reach steady states for well annealed samples in the pre-yield regime. For poorly annealed samples, where the sample undergoes driving induced annealing before reaching a steady state, we investigate the time taken for this steady state to be reached by similarly fitting the time-dependence of the energy to the form described above.

The times to steady state obtained from data in Fig. S14 and Fig. S15 are compiled in Fig. 3 (A,B,C) in the main manuscript, with a summary of the behaviour of f_{yield} with τ_p in Fig. 3 (D) in the main manuscript. In Fig. S16 below, we show additionally a comparison

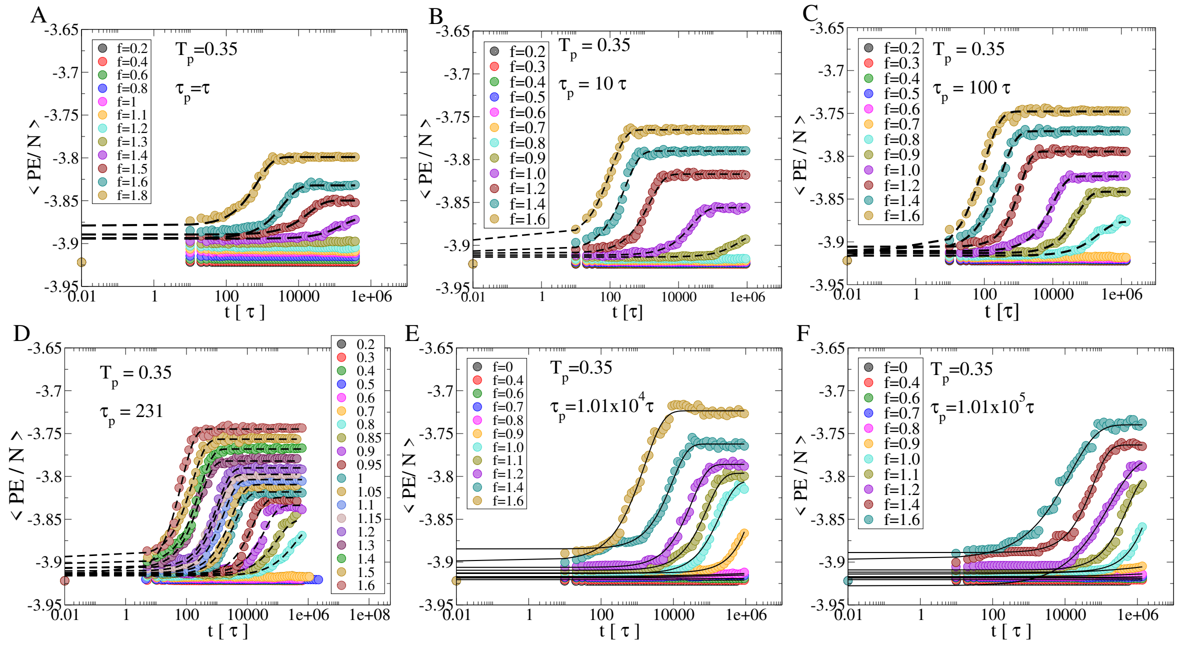

FIG. S14. Potential energy vs time, averaged over 8 independent simulations, starting from a well-annealed sample at $T_p = 0.35$ at 6 values of the persistence time, τ_p. Data are obtained from active dynamics simulations performed with a timestep of $dt = 0.01$. Dashed lines are stretched exponential fits to the data.

of the data in our work with recent work also investigating the role of persistence times on the value of f at which fluids arrest under active dynamics [18].

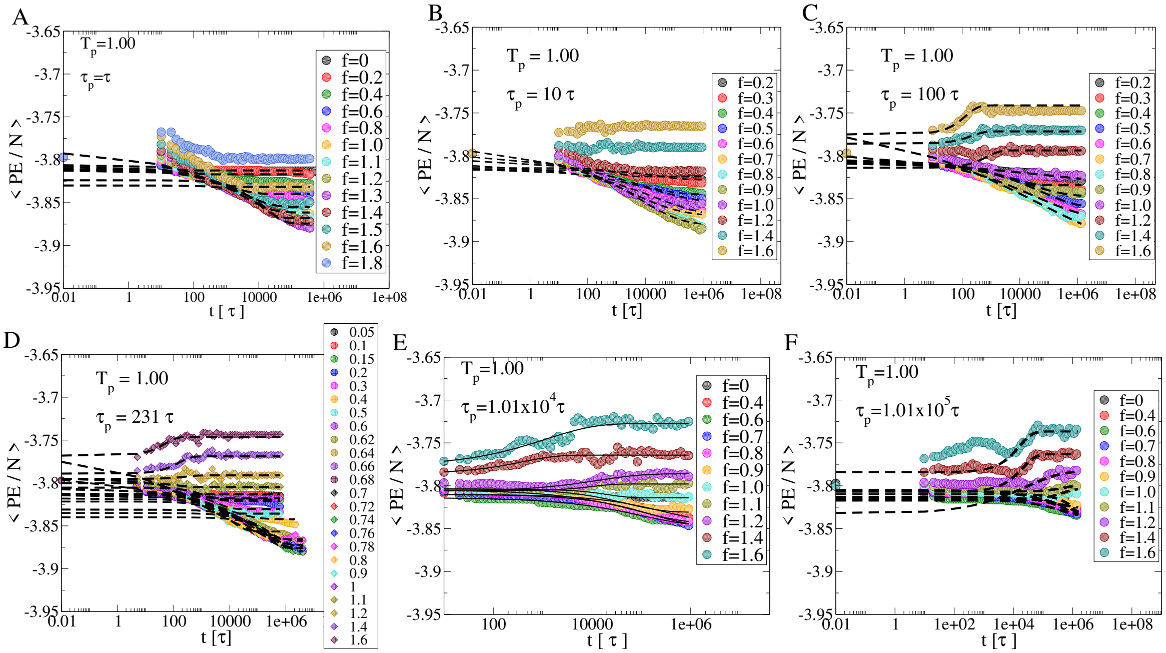

FIG. S15. Potential energy vs time, averaged over 8 independent simulations, starting from a poorly-annealed sample at $T_p = 1.00$ at 6 values of the persistence time, τ_p. Data are obtained from active dynamics simulations performed with a timestep of $dt = 0.01$. Dashed lines are stretched exponential fits to the data.

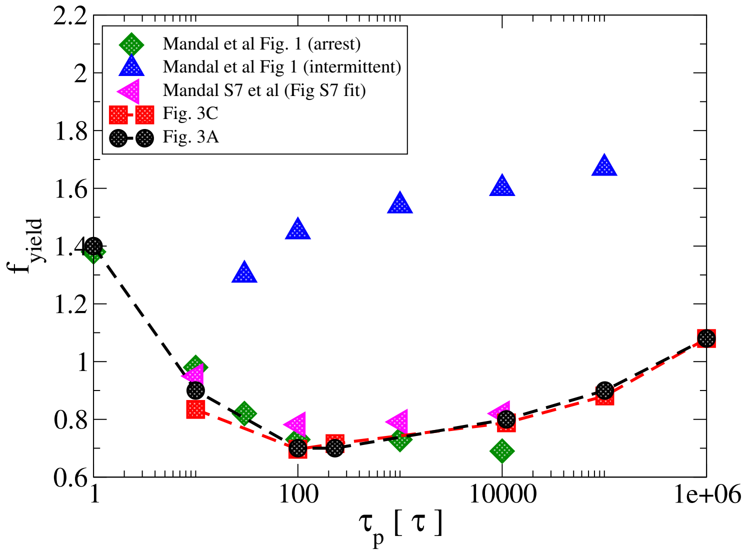

FIG. S16. The data from Fig. S7 of [18] are considered and the f at which the relaxation time, τ_α, can be extrapolated from the available data are noted. Further, if we consider an "iso-chrone" of $\tau_\alpha = 10^3$, we find that the $f_{\tau_\alpha=10^3}$ changes non-monotonically with τ_p.

S12. Confinement simulations

A. Confinement boundary implementation

In order to compute the wall interaction, we need to identify the vector from the centre of any particle, p, to the closest point, q, on the elliptical confinement boundary. We follow an iterative procedure with the following steps [19, 20]:

1. Pick a random initial a on the ellipse.
2. Compute the distance r_{pa} between p and a.
3. The circle of radius r_{pa} centred at p will intersect the ellipse at another point b.
4. Identify the midpoint a' between a and b.
5. Calculate $r_{pa'}$ and go to step 3.

In order to follow this procedure, we describe the relevant details from [20] for completeness:

$$\begin{aligned} x &= a\cos(t) \\ y &= b\sin(t) \end{aligned} \tag{S11}$$

We can approximate the curvature of the ellipse at a to a circle centred at ev_x, ev_y with radius $|\omega_a|$. The centres of curvature of the ellipse are obtained using

$$\begin{aligned} ev_x &= \frac{(a^2 - b^2)}{a}\cos^3(t) \\ ev_y &= \frac{(b^2 - a^2)}{b}\sin^3(t) \end{aligned} \tag{S12}$$

Vectors from the centre of curvature to a and to a', ω_a and $\omega_{a'}$ are separated by an arclength Δc. We can write:

$$sin\left(\frac{\Delta c}{|\omega_a|}\right) = \frac{\omega_a \times \omega_{a'}}{|\omega_a||\omega_{a'}|} \tag{S13}$$

One can then write

$$\begin{aligned} \frac{dc}{dt} &= \sqrt{\left(\frac{dx}{dt}\right)^2 - \left(\frac{dy}{dt}\right)^2} \\ \frac{\Delta c}{\Delta t} &= \sqrt{a^2\sin^2(t) + b^2\cos^2(t)} \end{aligned} \tag{S14}$$

We can then identify Δt as

$$\Delta t = \frac{\Delta c}{\sqrt{a^2 + b^2 - x^2 - y^2}} \tag{S15}$$

Finally, one can find the coordinates of a' as

$$\begin{aligned} x' &= a\cos(t + \Delta t) \\ y' &= b\sin(t + \Delta t) \end{aligned} \tag{S16}$$

Once a' has been identified, we can iterate the procedure till convergence is achieved. In Fig. S17, we show heatmaps of the distance computed from ellipses of different aspect ratio, including the symmetric circular case.

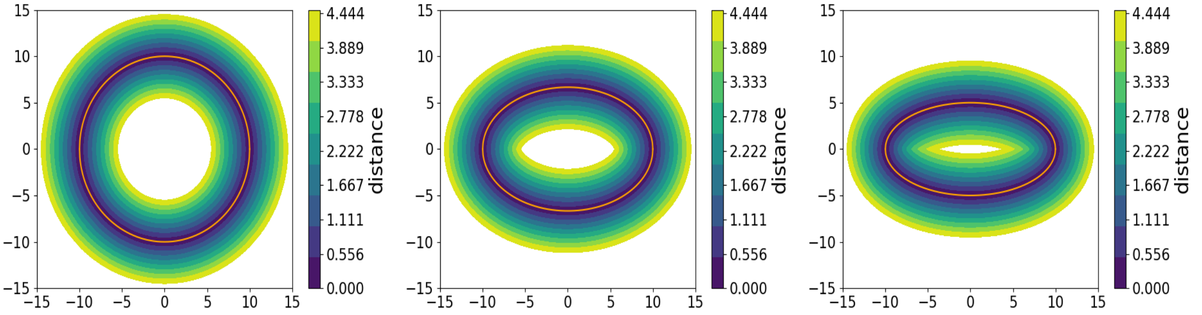

FIG. S17. Heat map of distance from the boundary of the ellipse, as computed by the optimisation procedure to identify the closest point on a given ellipse from a chosen query point.

B. Sample preparation in confinement

In order to prepare initial samples in confinement, we first thermally anneal the binary mixture at different simulation temperatures, T, shown in Fig. S18. Steady state configurations from the annealed trajectories are then subjected to an instantaneous thermal quench to $T_q = 0.0001$ and simulated for 5×10^4 MD steps to approximate a mechanically stable initial configuration.

C. Time evolution of energy in confinement

In Fig. S19 we show the time evolution of the average energy per particle for the two confinement geometries for both the well-annealed sample ($T_p = 0.4$) and the poorly annealed

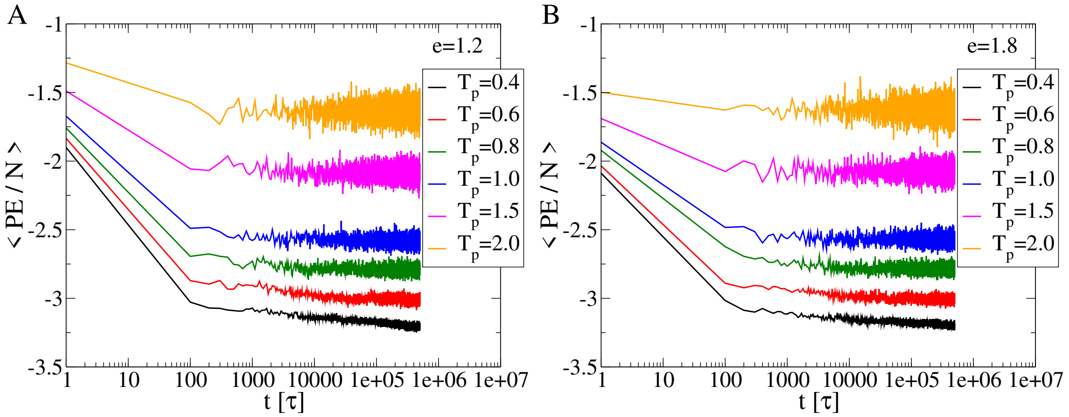

FIG. S18. Evolution of potential energy per particle over time for a system in two confinement geometries, $e = 1.2$ (left), and $e = 1.8$ (right) at different preparation temperatures, T_p.

one ($T_p = 1.0$). Well-annealed samples show a constant $\langle PE/N \rangle$ with increasing f for small f, followed by a steep rise as f is increased beyond the yield point. For the poorly annealed sample, the system undergoes further annealing with increasing f upto the yield point, with a corresponding monotonic decrease in $\langle PE/N \rangle$. The time taken to reach the steady state is longer close to the yield point, which we interpret will introduce difficulty in obtaining reliable estimates of the final steady state value of the potential energy, as can be seen for the case of $f = 0.6$ in Fig. S19 (a) and (c) and for the case of $f = 0.8$ in Fig. S19 (b) and (d).

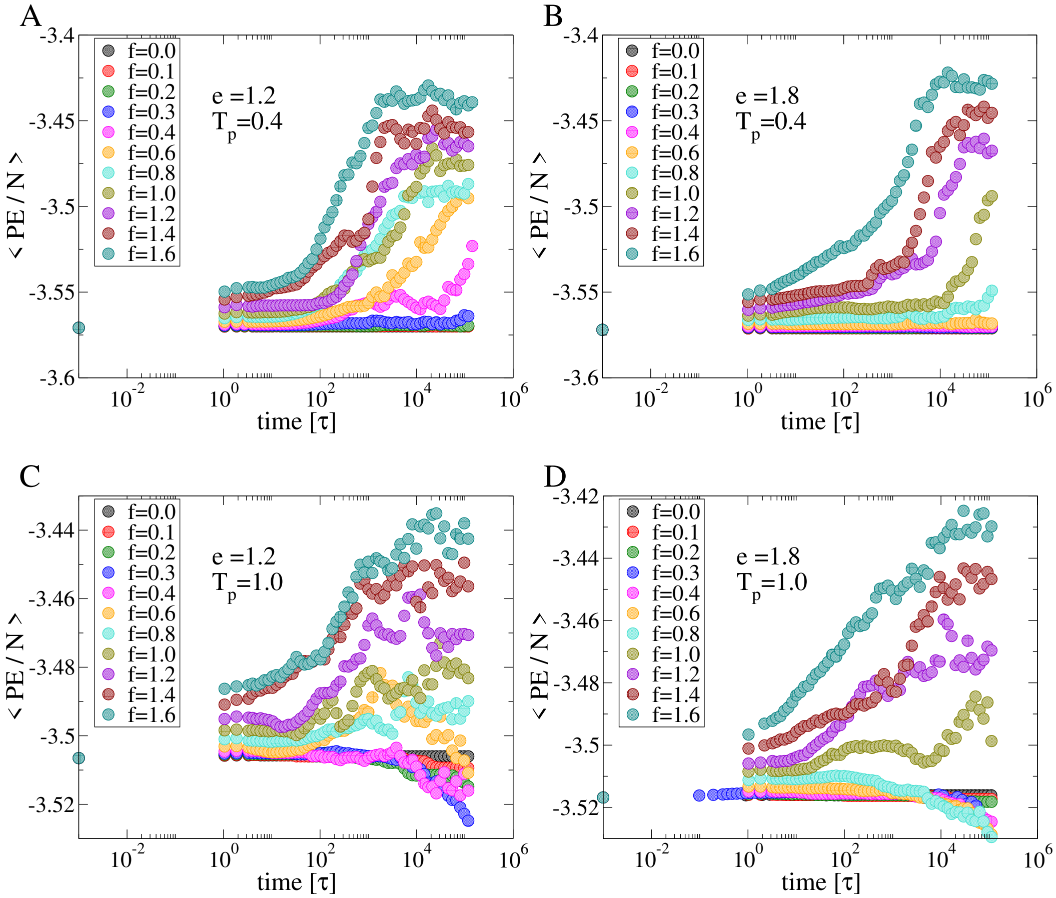

FIG. S19. Time evolution of average energy per particle at the values of e and T_p shown in the plots, averaged in logarithmically spaced bins. Curves reach a steady state for all values of active driving force, f, except those close to the respective transition values.

D. Particle density correlation and maps in confinement

We compute the histogram of particle relative densities with respect to their distance from the confinement boundary, with distance measured along the normal to the ellipse at closest approach between a given particle centre and the confinement boundary. This produces a wall-particle density correlation function, $g_w(r)$, from which one can straightforwardly obtain a total correlation function, $h_w(r) = g_w(r) - 1$, shown in Fig. S20, whose peak heights decay exponentially according to the real space form of the Ornstein-Zernike equation in 2D [21] in Eq. S17 below.

$$h(r) = \frac{Ae^{-r/C_l}}{r^{d-2}} \tag{S17}$$

We identify the peaks from the data and fit the peak height decay to Eq. S17 and extract the correlation lengthscale C_l shown in Fig. 4 (C) of the main manuscript. We consider the

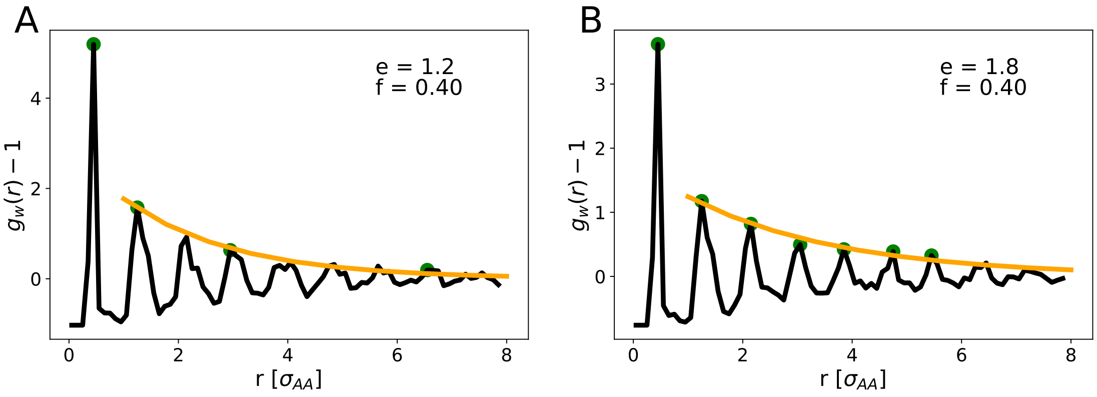

FIG. S20. The wall-particle pair correlation, $g_w(r) - 1$, with the peaks (green circles) fit to the form in Eq. S17 (orange line) , shown for $e = 1.2$ for one value of f (see figure text) in panel (A) and for $e = 1.8$ and the same value of f in panel (B).

density profiles in 2D, by averaging over independent simulations and snapshots separated by 10^6 MD steps, for the two geometries, shown in Fig. S21. One observes a switch in the density profiles from strongly patterned to more homogeneous across the yield point, which differs for the two geometries. We look at curvature along the lines discussed in [22], where

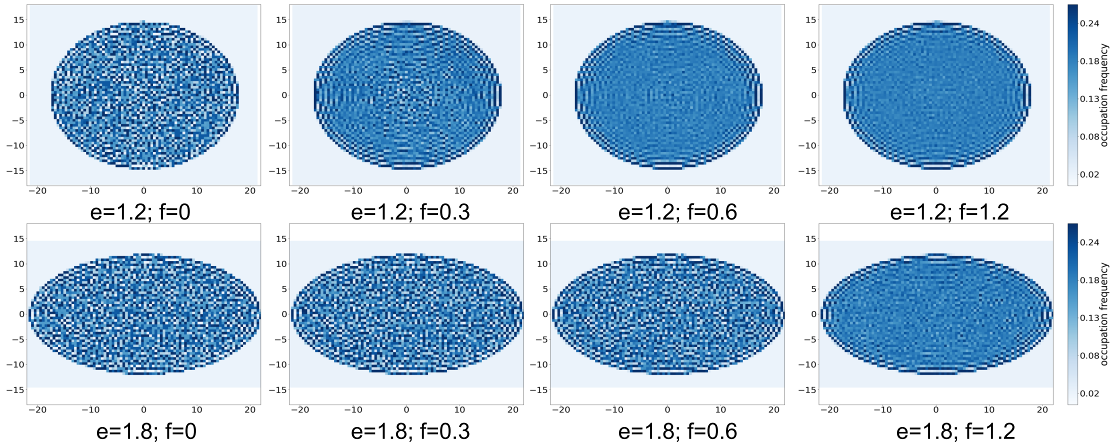

FIG. S21. 2D density profile for active dynamics simulations in confinement at 4 values of f.

the accumulation of non-interacting active particles at regions of high curvature, as well as changes in the alignment of motion as a function of curvature were investigated. We find that

high curvature causes disalignment of particle motion with respect to neighbours, preventing fluidisation in the higher aspect ratio geometry. In order to measure the alignment in motion, we consider the angle of displacement for particles, indexed i, within the first shell of the wall-particle density correlation function.

$$\omega_i = \tan^{-1}\left(\frac{y_i(t+\Delta t) - y_i(t)}{x_i(t+\Delta t) - x_i(t)}\right) \tag{S18}$$

Similarly, we consider the angles ω_j for displacements of particles, j, within the LJ cutoff of $2.5\sigma_{ab}$ $a, b \in [A, B]$ of i at t. The average of the difference in these angles gives a measure of how much the motion of i is aligned with the motion of its neighbours j over the time window $[t, t + \Delta t]$. We quantify this by measuring $\langle \cos(\Delta\omega_{ij} \rangle$, where the average is over multiple snapshots t and 8 independent runs.

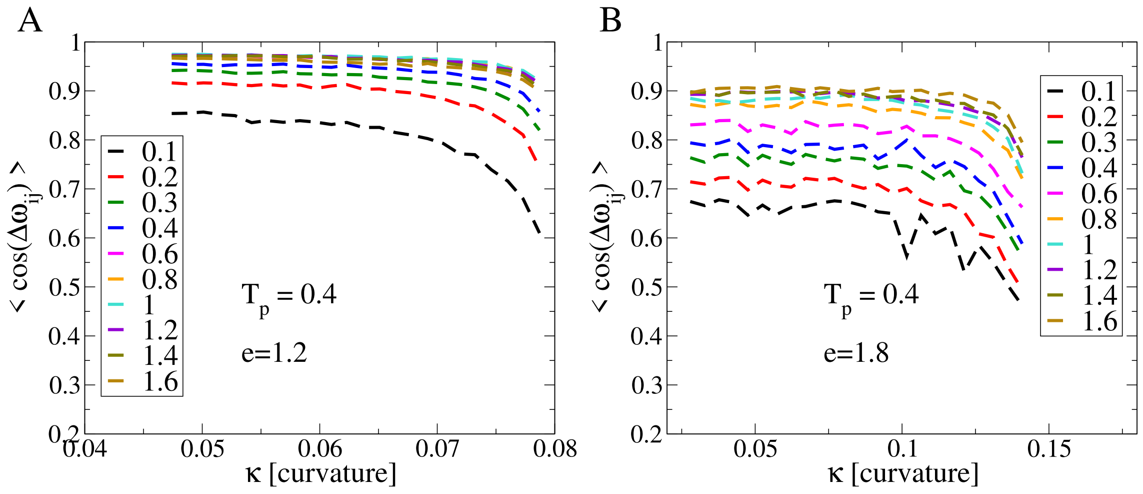

FIG. S22. The cosine of the relative orientation of motion of particles i with neighbours j (see text) $\cos(\Delta\omega_{ij})$, over a finite time window Δt for particles i close to the confinement boundary (left and middle panel). We observe that the alignment is high where the curvature of the boundary is low, and decreases as curvature increases. The change in alignment over the full range of curvature is summarised for different f in Fig. 4 in the main manuscript.

S13. Description of Supplementary Videos

Supplementary Video 1: Timelapse of trajectory for $N = 1000$ particles simulated in confinement in an ellipsoid of aspect ratio $e = 1.2$, with $f = 0.6$, showing large scale rearrangements involving coherent motion.

Supplementary Video 2: Timelapse of trajectory for $N = 1000$ particles simulated in confinement in an ellipsoid of aspect ratio $e = 1.8$, with $f = 0.6$, showing relative quiescence with vibrations of particle around their mean positions.

---

[1] Benedict Leimkuhler and Charles Matthews. Rational construction of stochastic numerical methods for molecular sampling. *Applied Mathematics Research eXpress*, 2013(1):34–56, 2013.

[2] Michael P Allen and Dominic J Tildesley. *Computer simulation of liquids*. Oxford university press, 2017.

[3] Carlos Villarroel and Gustavo Düring. Critical yielding rheology: from externally deformed glasses to active systems. *Soft Matter*, 17:9944–9949, 2021.

[4] Haiyan Xu, Juan Carlos Andresen, and Ido Regev. Yielding in an amorphous solid subject to constant stress at finite temperatures. *Physical Review E*, 103(5):052604, 2021.

[5] Daniel Bonn, Morton M Denn, Ludovic Berthier, Thibaut Divoux, and Sébastien Manneville. Yield stress materials in soft condensed matter. *Reviews of Modern Physics*, 89(3):035005, 2017.

[6] Thibaut Divoux, Catherine Barentin, and Sébastien Manneville. From stress-induced fluidization processes to herschel-bulkley behaviour in simple yield stress fluids. *Soft Matter*, 7(18):8409–8418, 2011.

[7] Thibaut Divoux, David Tamarii, Catherine Barentin, Stephen Teitel, and Sébastien Manneville. Yielding dynamics of a herschel–bulkley fluid: a critical-like fluidization behaviour. *Soft Matter*, 8(15):4151–4164, 2012.

[8] Chen Liu, Kirsten Martens, and Jean-Louis Barrat. Mean-field scenario for the athermal creep dynamics of yield-stress fluids. *Physical Review Letters*, 120(2):028004, 2018.

[9] Ruoyang Mo, Qinyi Liao, and Ning Xu. Rheological similarities between dense self-propelled and sheared particulate systems. *Soft Matter*, 16:3642–3648, 2020.

[10] Norihiro Oyama, Hideyuki Mizuno, and Atsushi Ikeda. Instantaneous normal modes reveal structural signatures for the herschel-bulkley rheology in sheared glasses. *Physical Review Letters*, 127(10):108003, 2021.

[11] Chen Liu, Ezequiel E Ferrero, Francesco Puosi, Jean-Louis Barrat, and Kirsten Martens. Driving rate dependence of avalanche statistics and shapes at the yielding transition. *Physical review letters*, 116(6):065501, 2016.

[12] Daniel Vågberg, Peter Olsson, and S Teitel. Universality of jamming criticality in overdamped shear-driven frictionless disks. *Physical Review Letters*, 113(14):148002, 2014.

[13] Gaurav Prakash Shrivastav, Pinaki Chaudhuri, and Jürgen Horbach. Yielding of glass under shear: A directed percolation transition precedes shear-band formation. *Physical Review E*, 94(4):042605, 2016.

[14] Steve Plimpton. Fast parallel algorithms for short-range molecular dynamics. *Journal of computational physics*, 117(1):1–19, 1995.

[15] Pallabi Das, HA Vinutha, and Srikanth Sastry. Unified phase diagram of reversible–irreversible, jamming, and yielding transitions in cyclically sheared soft-sphere packings. *Proceedings of the National Academy of Sciences*, 117(19):10203–10209, 2020.

[16] Saroj Kumar Nandi, Rituparno Mandal, Pranab Jyoti Bhuyan, Chandan Dasgupta, Madan Rao, and Nir S Gov. A random first-order transition theory for an active glass. *Proceedings of the National Academy of Sciences*, 115(30):7688–7693, 2018.

[17] Rituparno Mandal, Saroj Kumar Nandi, Chandan Dasgupta, Peter Sollich, and Nir S Gov. The random first-order transition theory of active glass in the high-activity regime. *Journal of Physics Communications*, 6(11):115001, 2022.

[18] Rituparno Mandal, Pranab Jyoti Bhuyan, Pinaki Chaudhuri, Chandan Dasgupta, and Madan Rao. Extreme active matter at high densities. *Nature communications*, 11(1):2581, 2020.

[19] L Maisonobe. Quick computation of the distance between a point and an ellipse, 2006.

[20] Carl Chatfield. A simple method for distance to ellipse. `https://chatfield.io/simple-method-for-distance-to-ellipse/.`, 2017.

[21] H Eugene Stanley. *Phase transitions and critical phenomena*, volume 7. Clarendon Press, Oxford, 1971.

[22] Yaouen Fily, Aparna Baskaran, and Michael F Hagan. Dynamics of self-propelled particles under strong confinement. *Soft matter*, 10(30):5609–5617, 2014.
